# Infectious disease outcomes associated with inadequate housing and access to healthy living practices in Australia: a systematic review

**DOI:** 10.1136/bmjph-2025-003531

**Published:** 2026-02-27

**Authors:** Kate Summer, Keerthi Anpalagan, Ingrid Stacey, Samantha Stiles, Rachel Burgess, Vicki Wade, Asha C. Bowen, Judith Katzenellenbogen, Rosemary Wyber

**Affiliations:** 1 Wesfarmers Centre for Vaccines and Infectious Diseases, The Kids Research Institute Australia, Nedlands, Western Australia, Australia; 2 Department of Infectious Diseases, Perth Children’s Hospital, Nedlands, Western Australia, Australia; 3 Cardiovascular Epidemiology Research Centre, The University of Western Australia, Nedlands, Western Australia, Australia; 4 Cardiology Population Health Laboratory, Victor Chang Cardiac Research Institute, Darlinghurst, New South Wales, Australia; 5 Menzies School of Health Research, Darwin, Northern Territory, Australia; 6 Yardhura Walani, Australian National University, Canberra, Australian Capital Territory, Australia

**Keywords:** Disease Transmission, Infectious, Data Collection, Public Health, Community Health, Program Evaluation

## Abstract

**Objectives:**

Inadequate housing and living conditions underpin significant health and wellbeing inequality in Australia, particularly for Indigenous people. This review aimed to define infectious disease (ID) outcomes used to measure the health impact of inadequate housing in Australia within a research context.

**Design:**

A systematic review of published studies following the Preferred Reporting Items for Systematic reviews and Meta-Analyses (PRISMA) guidelines.

**Data sources:**

Four online databases were searched in May 2024 with no date restrictions using terms relating to housing, ID and Australia. Results were screened in Covidence.

**Eligibility criteria:**

Studies were included if they measured both housing exposures and ID outcome variables among any population group in Australia. Both quantitative and qualitative designs were considered.

**Data extraction and synthesis:**

Data were extracted from 81 studies. Exposures and outcomes were classified according to the Healthy Living Practices (HLPs) and the International Classification of Primary Care (ICPC-2) body system classes, respectively. Acute infections and chronic post-infectious sequelae were considered ID outcomes. Descriptive statistics were calculated and ID-HLP associations were synthesised and narratively described. Methodological quality was assessed from both biomedical and Indigenous perspectives using relevant appraisal tools.

**Results:**

Most studies (79%) were published within the past 25 years and more than half (60%) involved remote-living Indigenous children and families. A total of 176 measured ID outcomes and 571 ID-HLP associations were tested. Exposures were frequently related to the negative effects of crowding (HLP5), unsafe wastewater treatment/drinking water (HLP3) and reduced capacity for washing people (HLP1). Skin, respiratory and digestive system infections were the most common ID outcomes, followed by eye and ear infections and post-infectious cardiovascular sequelae. Studies relied on intensive data collection methods and bespoke definitions.

**Conclusions:**

Substantial research has described ID outcomes associated with inadequate housing in Australia. However, high-quality evidence is lacking, and methodological heterogeneity between studies limits the synthesis and actionability of this work. We recommend prospective classification of housing exposures according to the HLPs and encourage the exploration of routinely collected primary care data. Agreed measurement approaches and data collection tools that are consistent with Indigenous Data Sovereignty principles would add value.

**PROSPERO registration number:**

CRD42024541393.

**WHAT IS ALREADY KNOWN ON THIS TOPIC**

- Adequate housing, in terms of quantity and quality, is a known social determinant of health and wellbeing. However, specific associations between housing and health can be challenging to define.
- Functional housing infrastructure underpins Healthy Living Practices (HLPs), such as washing hands, bodies and clothes, which have strong links to the prevention of infectious disease (ID).

**WHAT THIS STUDY ADDS**

- This systematic review applies the HLPs and the International Classification of Primary Care (ICPC-2) body system classes to classify and synthesise available information collected over decades of Australian research into ID outcomes associated with specific housing variables.

**HOW THIS STUDY MAY AFFECT RESEARCH, PRACTICE OR POLICY**

- Academic studies into Indigenous housing and health are inconsistent and have a limited influence on policy.
- Refining an accepted list of housing-associated ID outcomes recorded in primary care could streamline evaluation and monitoring within research, service provider and policy settings.

## Introduction

 Housing of a sufficient standard is foundational for leading a healthy life.^1^,^3^ Conversely, poor housing and living conditions are one of the primary mechanisms through which social and environmental inequities translate into inequalities in health and wellbeing.^2^ Inadequate housing and living conditions can affect health and wellbeing in a myriad of ways, but the association between crowded, under-resourced living conditions and infectious/communicable diseases (IDs) is best described.^4^,^7^

Australia is a wealthy country but income and wealth are not distributed equally.^8 9^ There are many, often marginalised, Australians who occupy substandard housing with little capacity to change or remedy their circumstances.^4 6 10^ In 2016, it was estimated that over one million Australians occupied housing considered to be in poor, very poor or derelict condition.^4^ In the 2019–2020 Survey of Income and Housing, 22% of social housing renters (representing 4% of total households in Australia)^11^ reported major structural problems in their current dwelling and low levels of housing satisfaction compared with private home owners.^12^ Chronic underinvestment in the social and affordable housing sector has converged with recent surges in market prices, significantly increasing the proportion of people experiencing housing stress.^13^ Irrespective of knowledge and awareness, reduced access to affordable, functional housing and infrastructure can hinder access to health-seeking behaviours, or Healthy Living Practices (HLPs),^14^ and thus capacities to achieve and maintain healthy lives.^15^,^17^ Young and older people, sole parent families, people with long-term illnesses and disabilities, migrants/refugees, the un/underemployed and, most strikingly, Aboriginal and Torres Strait Islander people, occupy most of Australia’s poor housing stock.^4 18^ These cohorts are doubly forced to tolerate pre-existing socioeconomic disadvantage *and* the impacts of inadequate housing.^4^

The impacts of inadequate housing in Australia are especially profound for remote-living Aboriginal and Torres Strait Islander (hereafter respectfully referred to as Indigenous) people and communities. This is a result of ongoing colonisation and systemic racism, geographic isolation, socioeconomic marginalisation, under-representation in data collection and unresponsive housing policy.^1019^,^21^ Disproportionately high rates of preventable infectious and chronic diseases and gaps in life expectancy are direct consequences.^6^ The infectious disease (ID) burden experienced by Indigenous people is more than three times that of the non-Indigenous population, and sequelae arising, including loss of hearing and vision, acute rheumatic fever (ARF) and rheumatic heart disease (RHD), are among the highest in the world.^22^

Tools for routinely monitoring the longitudinal health impacts of inadequate housing and living conditions, as well as improvement initiatives, are needed to identify priorities and effective housing policy options.^23 24^ Such monitoring tools could, and should, exist beyond the research context.^23^ However, their development is stymied by fragmented understanding, delays in data collection and inconsistent measurement of exposures and outcomes. This review aims to identify the ID outcomes that have been used to measure the impact of inadequate housing on health in the Australian context. We sought to classify housing exposures and ID outcomes by predefined classification frameworks (the HLPs^14^ and the International Classification of Primary Care^25^ [ICPC-2] body system classes, respectively) to synthesise this body of work. Improved understanding of housing-associated ID outcomes will inform the development of agreed research approaches and robust tools for monitoring.^23^

## Methods

A systematic review was conducted following the Preferred Reporting Items for Systematic reviews and Meta-Analyses (PRISMA) guidelines^26^ (PROSPERO registration CRD42024541393). It was co-designed and co-authored by Indigenous (VW, RB) and non-Indigenous (KS, KA, IS, SS, JK, AB, RW) collaborators in response to research priorities established by the Indigenous Governance Council (IGC) for a National Health and Medical Research Council-funded programme of work related to Indigenous environmental health. Ways of working on this review were aligned with the CONSIDER statement^27^ (online supplemental material).

### Search and screening

Online databases Scopus, PubMed and Informit were searched in May 2024 using terms relating to housing, ID and Australia (online supplemental material). A targeted search in MEDLINE (a subset of PubMed) was also undertaken using MeSH (Medical Subject Heading) terms (online supplemental material). Results were limited to articles published in English with no date restrictions. References were catalogued in Covidence^28^ and independently screened by two reviewers (KS and KA). Any conflicts were discussed and resolved by a third reviewer (RW). Automatic removal of duplicates was supplemented by manual checking. Quantitative and qualitative study designs were considered.

A population, exposure, outcome (PEO) framework was used to define study inclusion/exclusion criteria.^29^ The following criteria were used to assess the suitability of articles for inclusion:

1. The study population was based in Australia (Indigenous and/or non-Indigenous), of any age.
2. Exposure measure/s were related to the HLPs (described in the online supplemental material), housing or environmental health variables.
3. Outcome measure/s included at least one ID diagnosis.

Some topics were excluded to ensure the relevance of reviewed studies. Exclusion criteria are detailed in the online supplemental material. For example, studies investigating different housing arrangements (eg, rental vs home ownership), homelessness and institutions (eg, prisons and early education centres) were excluded. Both housing conditions and ID outcomes must have been measured, not only discussed.

### Data extraction and analysis

Data were extracted into a pre-designed spreadsheet and analysed in Microsoft Excel. One reviewer (KS) completed 100% of data extraction, with 25% independently checked by a second reviewer (KA). Metadata included study design, location information (community details, jurisdiction and classification of remoteness according to the Modified Monash Model (MMM),^30^ population information (age and population group), sources of data for ID outcomes and HLP exposures, strength of association (if reported), laboratory confirmation of diagnoses and the data collection period).

In the absence of universally accepted classification systems for housing exposures and ID outcomes, we applied existing frameworks with greatest relevance to Indigenous communities and most applicable to the development of a monitoring tool. Housing exposures and ID outcomes were recorded as described in each article, then classified according to the HLPs^14^ and ICPC-2 body system classes,^25^ respectively. Details on HLP and ICPC-2 frameworks and classification decisions are provided in the online supplemental material. We also classified the sources of ID outcome data (eg, primary care data, hospital data, researcher collected, self-reported, mixed, notifiable disease registries). The effect of each tested ID-HLP association was coded as 0, not mewellasured; 1, measured and positive association found; 2, measured and no association found; or 3, measured and negative association found. Pivot tables were then used to tabulate the number of times respective ID-HLP associations were measured and the number of times that measured associations were reported as being positive and/or significant. Descriptive statistics relating to study characteristics were calculated and narratively described.

### Quality assessment

The methodological quality of included studies was determined using Joanna Briggs Institute critical appraisal tools (JBI tools) appropriate to each study design (https://jbi.global/critical-appraisal-tools). In parallel, quality assessment of applicable studies was conducted using the Aboriginal and Torres Strait Islander Quality Appraisal Tool (QAT),^31^ which enables an assessment of study quality and value with respect to the cultural appropriateness of research. Scoring was undertaken by two reviewers with oversight from experts in respective tools (online supplemental material).

### Patient or public involvement

This article is a review of existing literature synthesising previously published data. Patients and/or the public were therefore not directly involved. However, we recognise the value of patient and public involvement in the dissemination of this article and in shaping further research and development priorities. Patient and public involvement will occur in the next phases of our work informed by this systematic review.

## Results

### Search and screening

**Figure 1:**
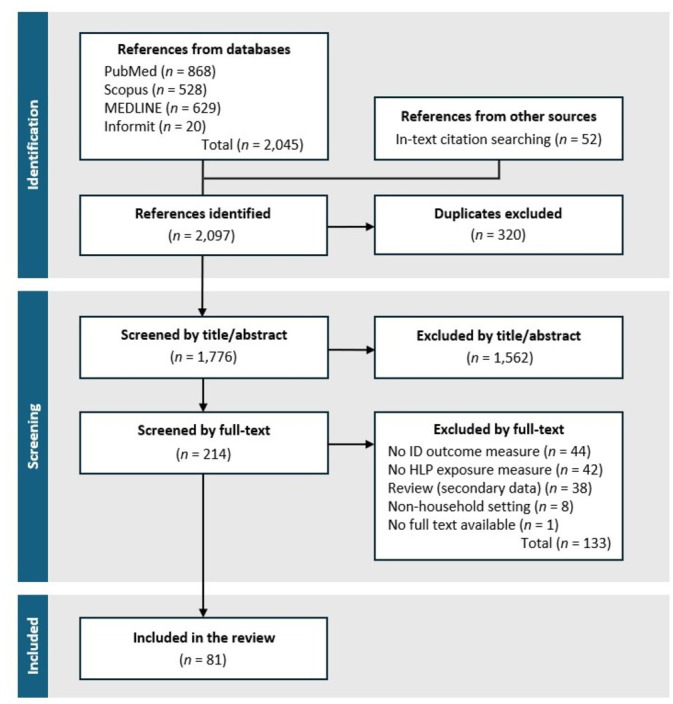
Flow chart of search and screening record. HLP: Healthy Living Practice or housing-related exposure measure; ID: infectious disease.

Of the 2097 references identified, 320 were duplicates and 1562 were removed at title/abstract level (figure 1). This left 214 for full text review, of which 81 met inclusion criteria (figure 1). The leading reason for full-text exclusion was measurement of either housing/HLP exposures or ID endpoints but not both (figure 1). Included studies are detailed in the online supplemental material. Many studies that were excluded from this review measured the functionality of housing against HLP criteria but not the associated ID outcomes,^32^,^34^ and vice versa.^35 36^

### Overview of included studies

Most studies were cohort (n=31, 38%) or cross-sectional (n=25, 31%) designs (table 1). Other designs included case-control studies, case studies, ID modelling/simulation studies, mixed-method studies, one randomised controlled trial and five qualitative studies (table 1). Sixty-four studies (79%) were published after the year 2000 (table 1). Recent studies (2015–2024) used the most diverse range of sources for obtaining data on ID outcomes, with an increased focus on modelling/simulation studies, notifiable disease registries and self-reported data (tables 1 and 2).

**Table 1:** Summary of characteristics of the 81 studies reviewed by sources of data used to measure infectious disease (ID) outcomes in response to housing/Healthy Living Practice (HLP)-related exposures

Study characteristics	Source of health (ID outcome) data								Overall	
	Hospital data	Linked/public data*	Mixed sources	Model parameters	Notification data	Primary care	Researcher collected	Self-reported	No. of studies	% of total (n=81)
Study design										
Case control					4	1	2		7	9
Case study	1						1		2	2
Cohort	2		7		3	1	12	6	31	38
Cross-sectional		1	4		8	3	5	4	25	31
Mixed method			3					1	4	5
Model				6					6	7
Qualitative								5	5	6
Randomised controlled trial								1	1	1
Total	3	1	14	6	15	5	20	17	81	100
% of total (n=81)	4	1	17	7	19	6	25	21	–	–
Publication date range										
Pre-2000	1		5		2	1	8		17	21
2000–2014	1		3	1	4	2	9	9	29	36
2015–2024	1	1	6	5	9	2	3	8	35	43
Study population										
Aboriginal/Torres Strait Islander†	2	1	10	5	2	2	16	10	48	60
Mixed population					4		2	2	8	9
Not specified	1		4	1	9	3	3	4	25	31
Age group										
Children/adolescent (0–17 y)	2		5	1	2	1	14	8	33	41
Adult (>17 y)			1	1	1		2	2	7	9
Mixed	1		8	3	11	3	4	7	37	46
Not specified		1		1	1	1			4	5
Location/setting										
*Jurisdiction*										
New South Wales	1		2		2	1		3	9	11
Queensland (Qld)			3		3		5	2	13	16
Northern Territory (NT)			7	1	2	1	9	4	24	30
Western Australia (WA)	1	1			2		4	1	9	11
Northern WA, Qld and NT						1			1	1
South Australia (SA)					1	1	1	4	7	9
Victoria (Vic)	1		1		1	1	1	1	6	7
NA				3					3	4
National	1			2	4			2	9	11
*Modified Monash Model (MMM)*										
MMM 1–3 (metro-large towns)	1		3		4	1	5	7	21	26
MMM 4–5 (small-medium rural towns)			1		2		2		5	6
MMM 6–7 (remote/very remote)	2	1	9	4	2	3	11	3	35	43
Mixed locations			1	2	7	1	2	7	20	25
Housing exposure data source										
Self-reported	1	1	6	1	6	2	6	12	35	43
Researcher collected			7		3	2	13	4	29	36
Notification data					4				4	5
Linked/publicly available data			1		1	1			3	4
Mixed					1			1	2	2
Model				5					5	6
Patient medical records	2						1		3	4
Laboratory-verified ID diagnoses										
Yes	3		7		15	2	12	1	40	49
No		1	7	3		3	7	16	37	46
NA				3			1		4	5

* Linked data sources other than notifiable disease registries or hospital admission data, including data from the Australian Bureau of Statistics, Australian Institute of Health and Welfare etc.

† Three of these studies also reported data on a small non-Indigenous population, but the study was focused on the Aboriginal and/or Torres Strait Islander community.

More than half of studies (n=48, 60%) investigating associations between housing and ID outcomes involved Indigenous people (tables 1 and 2). Thirty-five studies (43% of all studies and 73% of studies involving Indigenous people) were set in remote/very remote (MMM 6–7) communities predominantly located in the Northern Territory and Western Australia (table 1). Population groups of interest were mostly children/adolescents (0–17 y) (n=33, 41%) or families/mixed age groups (n=37, 46%) (table 1). In total, there were 176 measured ID outcomes and 571 ID-HLP associations tested across all 81 studies (table 2).

**Table 2:** Overview of the number of associations tested between infectious disease (ID) outcomes and housing variables classified by the Healthy Living Practices (HLPs). ID outcomes, ID outcome data sources, and HLP exposures have been retrospectively classified for consistency, but this dataset remains inherently difficult to synthesise. Listed in descending order of number of ID-HLP associations tested.

First author surname	Study population	ID outcome data source	No. of IDs	No. of HLPs	ID-HLP Assoc’s tested
McDonald (2010)^37^	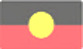	HD+SR	9	7	**63**
Bailie (2012)^38^	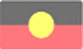	SR	5	10	**50**
Bailie (2010)^39^	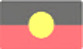	SR	5	10	**50**
Melody (2016)^68^	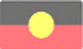	LD	8	6	**48**
Harris (1990)^69^	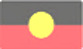	HD+RC	7	6-May	**40**
Ralph (2022)^70^	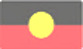	PC	13	4-Jan	**31**
Edwards (1970)^71^	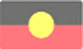	SR+RC	5	5	**25**
McDonald (2009)^72^	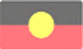	SR	3	7-Jun	**19**
Memmott (2022)^5^	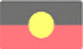	SR+PC	10	4-Jan	**19**
Chakraborty (2021)^73^	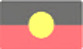	SR	3	5	**15**
Dossetor (2017)^74^	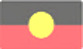	HD	6	2	**12**
Leach (2016)^75^	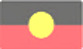	RC	6	2	**12**
Singleton (2014)^76^	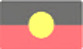	RC	3	4	**12**
Lansingh (2010)^40^	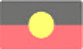	RC	1	9	**9**
Najnin (2014)^77^		SR	3	3	**9**
Wong (2002)^78^	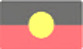	RC	3	3	**9**
Foster (2021)^79^	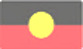	NDR+LD	4	2	**8**
Tedesco (1980)^80^	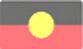	RC	1	8	**8**
Andersen (2018)^51^	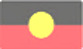	SR	1	7	**7**
Ewald (2003)^41^	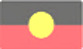	RC	1	6	**6**
Andersen (2016)^17^	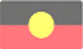	SR	5	1	**5**
Bailie (2005)^42^	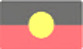	SR+PC	1	5	**5**
Kerrigan (2021)^81^	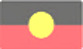	SR	1	5	**5**
Milazzo (2017)^82^		NDR	2	2	**4**
Williams (2015)^83^		RC	1	4	**4**
Williams (2016)^84^		NDR	1	4	**4**
Wozniak (2022)^85^		PC	4	1	**4**
Cooper (1986)^86^	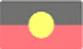	RC	1	3	**3**
Hempenstall (2021)^87^	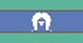	RC	3	1	**3**
McDonald (2008)^88^	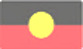	SR+RC	3	1	**3**
Merianos (1995)^89^	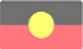	HD+NDR	1	3	**3**
Moffatt (2020)^90^		NDR	1	3	**3**
Peach (1997)^91^		RC	1	3	**3**
Sinclair (2010)^92^		PC	3	1	**3**
Boreham (1986)^93^		SR+RC	1	2	**2**
Hanna (1996)^94^	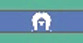	RC	1	2	**2**
Harris (1984)^95^	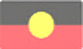	HD	1	2	**2**
Heyworth (2003)^96^		SR	1	2	**2**
Heyworth (2006)^97^		SR	1	2	**2**
Hodgetts (2022)^98^	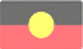	NDR	1	2	**2**
Kaminski (1977)^99^	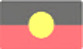	RC	2	1	**2**
La Vincente^100^	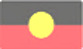	SR+RC	2	1	**2**
Massey^101^	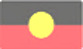	SR	1	2	**2**
McDonald^102^	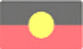	RC	2	1	**2**
McDonald^103^	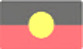	SR+RC	2	1	**2**
Pearce^104^	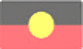	RC	1	2	**2**
Potter^105^		SR	1	2	**2**
Ratnaike^106^	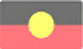	PC	1	2	**2**
Shattock^107^	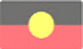	NDR	1	2	**2**
Spurling^50^	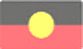	RC	1	2	**2**
Tenkate^108^		NDR	1	2	**2**
Unicomb^109^		NDR	1	2	**2**
Wright^110^		NDR	1	2	**2**
Zajaczkowski^111^		NDR	1	2	**2**
Akter^112^		NDR	1	1	**1**
Brown^113^		SR+RC	1	1	**1**
Carcione^114^		NDR	1	1	**1**
Carver^115^		NDR	1	1	**1**
Chen^116^		SR	1	1	**1**
Chisholm^117^	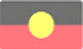	M	1	1	**1**
Hall^118^	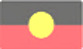	SR	1	1	**1**
Hui^119^	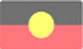	M	1	1	**1**
Inglis^120^	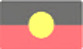	NDR	1	1	**1**
Jacoby^121^	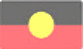	RC	1	1	**1**
Looker^122^		PC	1	1	**1**
Marshall^123^		NDR+RC	1	1	**1**
May^124^		NDR	1	1	**1**
McBride^125^		SR+RC	1	1	**1**
Meloni^126^	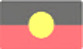	RC	1	1	**1**
Mishra^127^		M	1	1	**1**
Murray-Smith^128^		NDR	1	1	**1**
O'Toole^129^		SR	1	1	**1**
Oguoma^130^	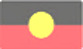	SR	1	1	**1**
Rodrigo^131^		SR	1	1	**1**
Schnagl^132^		RC	1	1	**1**
Schrieber^133^	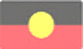	RC	1	1	**1**
Sordo^134^		NDR	1	1	**1**
Speare^135^		RC	1	1	**1**
Tellioglu^136^	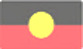	M	1	1	**1**
Vino^137^	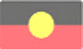	M	1	1	**1**
Williams^138^		HD	1	1	**1**
**Total**			**176**		**571**

Abbreviated infectious disease (ID) outcome data sources: HD: hospital data, LD: linked/public data, M: model, NDR: notifiable disease registry, PC: primary care, RC: researcher collected, SR: self-reported

Flag symbols are used to represent Aboriginal or Torres Strait Islander populations. Other study populations were mixed groups or unspecified.

### ID outcomes

ID outcome data were largely collected directly by researchers (n=20, 25%) (eg, microbiological swabbing), self-reported by participants or primary carers in questionnaires or focus groups (n=17, 21%), or obtained from notifiable disease registry databases (n=15, 19%) (table 1). Rarely were hospitalisation (n=3, 4%) or primary care records (n=5, 6%) used as sole sources of ID outcome data (tables1  2). Six studies (7%) made use of mathematical models to simulate associations using pre-determined ID parameters (tables1  2). A further 14 studies (18%) incorporated multiple data sources for measuring ID outcomes (tables 1 and 2). For example, researcher collected plus self-reported data or researcher collected plus hospital admission data; self-reported plus primary care data or self-reported plus hospital admission data (table 2). Actual descriptions of ID outcome data sources/data collection methods differed and were classified as required. Diagnoses were laboratory confirmed in less than half of studies (n=40, 49%) (table 1).

**Figure 2:**
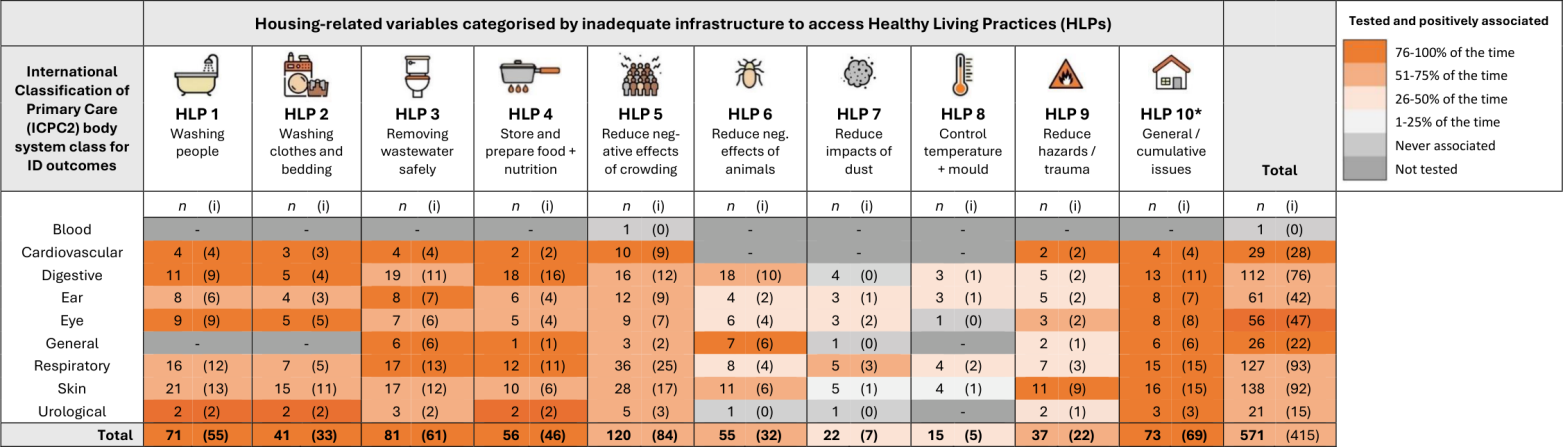
Counts (n) of infectious disease (ID) outcomes by International Classification of Primary Care (ICPC2) body systems measured in studies which also measured exposure to housing-related variables. Housing variables categorised by HLPs underpinned by relevant housing infrastructure. *n* = number of times the ID-HLP association was measured; values in brackets (i) indicate the number of times the measured association was reported as being positively associated and/or significant. Shading intensity is based on the number of positive associations relative to the number of associations tested (i/*n*×100). Data drawn from 81 reviewed studies using 176 ID outcomes testing 571 ID-HLP associations in total. *HLP10 was codified by the authors of this review.

Terminology used to describe the 176 ID outcomes varied and not all were unique conditions (approximately 80 unique conditions). ID outcomes affecting the skin, respiratory and digestive systems were most frequently investigated (measured 138, 127 and 112 times, respectively) (figure 2). ID outcomes relating to the ear and eye were also common (measured 61 and 56 times, respectively) (figure 2). ID outcomes affecting the cardiovascular and urological systems, and blood were investigated least (29, 21 and 1 times, respectively) (figure 2). Frequently investigated specific diagnoses included bacterial skin infection/skin sores (22 studies), gastroenteritis (15 studies), otitis media or ear infection (14 studies), scabies (12 studies), ARF or RHD (10 studies) and trachoma (nine studies). Respiratory infections included influenza (eight studies), pharyngitis (six studies) and pneumonia (three studies) and ‘respiratory infection’ without specific diagnoses (17 studies). Studies involving non-Indigenous or unspecified populations almost entirely investigated outbreaks of food poisoning (eg, salmonellosis, *Campylobacter* infection), mosquito-borne diseases (eg, dengue fever) or pandemic respiratory infections (eg, COVID-19, influenza). ID outcomes affecting the skin, cardiovascular, ear, eye, and urological body systems were investigated exclusively in Indigenous communities. Model-based studies were usually parameterised to model outcomes for an influenza-like illness.

### Housing/HLP exposures 

Seven studies used the HLPs as the original framework for measuring housing-related exposures^537^,^42^ (representing less than 9% of all studies and 15% of studies involving Indigenous people). Other housing-related exposures were study-specific and defined by the original researchers involved. Classification of housing exposures according to HLPs at our discretion was therefore required for most studies. There were no competing classification systems used to cover a range of housing-related variables other than the HLPs.

Most housing/HLP exposure data was self-reported in questionnaires or focus groups (n=35, 43%), or collected by researchers (n=29, 36%) in the form of a physical home assessment (eg, infrastructure surveys) or sampling (eg, from water, people, or pets) (table 1). Other sources of housing data, such as notifiable disease databases, other linked/publicly available data, patient medical records and predetermined model parameters provided information on household size/crowding (HLP 5) or sources of foodborne ID outbreaks/capacity to prepare food safely (HLP 4) as indicators but not direct measurements of the functional state of housing. As with ID outcomes, the terminology and methodologies used to describe similar housing exposures varied substantially.

Among the 571 ID-HLP associations tested, the most frequently measured housing/HLP exposures were related to the negative effects of crowding (HLP5; measured 120 times), ineffective wastewater removal/treatment (including access to safe drinking water) (HLP3; measured 81 times) and inadequate access to washing people (HLP1; measured 71 times) (figure 2). General or cumulative housing problems (HLP10) were measured 73 times (figure 2). Exposure to dust (HLP7) and inability to control temperature of the living environment (including mould) (HLP8) were measured least (figure 2).

### ID-HLP associations

The greatest range of ID-HLP associations (504 out of 571) were tested among studies prioritising Indigenous people; 23 studies tested five or more ID-HLP associations and 22 of these involved Indigenous people. Studies involving non-Indigenous, non-specified or mixed groups of people investigated fewer associations (table 2).

Inadequate access to water for hygiene and sanitation relating to HLPs 1–3 was most frequently tested in association with ID outcomes affecting the skin, respiratory and digestive systems (figure 2). Nutrition and the capacity to store and prepare food (HLP4) was often tested in association with digestive and respiratory infections (figure 2). Crowding (HLP5) was most frequently tested in association with IDs affecting the respiratory, skin, digestive, ear and cardiovascular systems (figure 2). Studies investigating the negative effects of animals, insects and vermin (HLP6) usually measured associations between digestive infections and dogs/cats; skin infections and dogs; respiratory infections and vermin; eye infections and flies; and general infections and mosquitoes. Exposure to hazards that cause physical trauma (HLP9) was usually tested in association with skin infections (figure 2).

Across all ID outcomes, access to HLPs 1–5 (washing people, washing clothes and bedding, removing wastewater, food and nutrition, crowding) was most often positively associated (76–100% of the time) (figure 2). The negative impact of animals/insects/vermin (HLP6) and rubbish (HLP9) was positively associated with ID outcomes 51%–75% of the time (figure 2). Exposure to dust (HLP7) was positively associated with respiratory ID outcomes (figure 2). There were few positive associations between capacities to control the temperature of the living environment (HLP8) and ID outcomes (figure 2). General or cumulative housing problems (HLP10) were almost always positively associated with ID outcomes (95% of the time). There were no negative associations ie, where lack of access to housing infrastructure/HLPs improved ID outcomes.

### Quality assessment

Seventy-three studies (90%) were eligible for appraisal using JBI tools, and 40 studies (49%) were eligible for appraisal using the Aboriginal and Torres Strait Islander QAT. All scores and reasons for reduced scores are supplied in the online supplemental material. Overall, the included studies were acceptable for the purpose of this review. However, methodological quality varied greatly from both biomedical and Indigenous perspectives, and few studies scored highly against both sets of tools. Studies assessed against QATs were published between 1970 and 2023. More recent studies reported checklist items in greater detail, which reflects improvements in research standards (and/or reporting) since the introduction of the JBI tools (early 2000s), the Aboriginal and Torres Strait Islander QAT (2020) and the Consolidated Criteria for Strengthening Reporting of Health Research Involving Indigenous Peoples (the CONSIDER statement, 2019).

## Discussion

This systematic review of 81 published studies identified a range of ID outcomes used to measure the health impact of inadequate housing in Australia within a research context. Our analysis showed that ID outcomes affecting the skin, respiratory and digestive systems have been most frequently investigated and positively associated with housing/HLP variables, followed by infections and chronic sequelae affecting the ear, eye and cardiovascular systems. In the Australian research context, there has been a specific focus on skin sores, gastroenteritis, general respiratory infections, otitis media, scabies, ARF/RHD and trachoma.

The ID-HLP associations tested were largely expected and pragmatic choices. A focus on access to safe water for drinking, sanitation and hygiene (ie, HLPs 1–3 or safe WASH principles) and sufficient housing stock to reduce the negative effects of crowding (ie, HLP5) reflects international community development priorities^3 43^ and the state of knowledge surrounding ID transmission and infection dynamics. Improvements in housing infrastructure with resultant improvements in capacities for HLPs 1–3 and HLP5 are likely to be beneficial, especially for skin, respiratory and digestive ID outcomes. General or cumulative housing issues (HLP10) were also frequently described and positively associated with ID outcomes but could not be meaningfully disaggregated or classified according to respective HLPs; this highlights the limited relevance of concepts such as ‘inadequate housing’ and ‘housing improvement’ if they cannot be practically linked to specific infrastructure and behaviours that underpin health and wellbeing outcomes.

The sources of data, and their inherent scale and reliability, also varied among the reviewed studies requiring a nuanced approach to interpretation. Most reported housing-associated ID outcomes are common, not severe/requiring hospitalisation and usually diagnosed and managed in local clinics.^44 45^ By contrast, hospital admissions and notifiable disease registries capture more severe, less frequent presentations and are likely to significantly underestimate primary care concerns.^46 47^ A more accurate picture of housing-associated ID outcomes is likely to be captured by self-reported and researcher collected data and by local clinics (primary care).^48^ If well recorded, routinely collected data have the potential to be extremely valuable for secondary use in monitoring the impacts of housing on health at a community scale (without additional research expense or intensity), they continue to be under used.^49^

Substantial research during the past 25 years has focused on the poor condition of housing and health impacts for Indigenous children and families living in remote areas of northern Australia. There are fewer but equally relevant studies describing the health impacts of inadequate housing for urban living Indigenous people.^17 50 51^ Research involving non-Indigenous Australians (or non-specified groups) tended to examine selected ID outcomes related to unusual circumstances (eg, epidemics, pandemics or outbreaks of food poisoning). This is in contrast to the diverse and often normalised diseases of poverty^52^ that have been used to measure the health impact of substandard housing for Indigenous people (eg, skin infections and RHD). The number of associations tested was also far greater in studies involving Indigenous compared with non-Indigenous Australians. This suggests that Indigenous housing improvement remains unaddressed nationally and that there has been a high burden of research with little translation to effective policy or service.

This review highlights the tensions between effectiveness/scientific rigour vs appropriateness/feasibility (ie, levels of evidence)^53^ and between perceptions of methodological quality (ie, biomedical vs cultural) in the academic study of associations between housing and health in Australia. Most reviewed studies were uncontrolled descriptive studies, likely due to the obvious ethical constraints in affording improvements in housing and living conditions to one group and not another (ie, randomised or non-randomised controlled interventions/trials). Models/simulation studies and mixed methods approaches may be increasingly prioritised to reduce the burden of on-ground research but incorporate different levels of evidence and therefore do not sit at a specific level within a hierarchy of evidence. Overall, future studies should be targeted to address specific knowledge gaps and should strive for quality from both biomedical and cultural perspectives, with a view to developing the best possible collective evidence base. Consistent and comparable approaches are better positioned to influence decision-making than smaller, sporadic studies with bespoke definitions and heterogenous methodologies. However, whether decisions are made on the basis of evidence is not guaranteed and will be invariably shaped by the wider ecology of policy.^54^

### Implications and recommendations

ID outcomes identified in this review are similar to the housing-associated ID indicators used internationally by the WHO^3 43^ (diarrhoeal disease, acute respiratory infections, soil-transmitted helminthiases) as well as in international research from Aotearoa New Zealand^55^ (enteric, respiratory, skin and eye infections) and Canada^56 57^ (respiratory and skin infections). Some similar indicators have been adopted in Australia, including by the Productivity Commission^21^ (intestinal infections, influenza and pneumonia, acute upper respiratory infections, bacterial diseases, trachoma, scabies, ARF/RHD, acute hepatitis) and in the 10-year review of the *Housing for Health* programme in New South Wales^58^ (skin, respiratory, ear and gastrointestinal infections). However, there is evidently no broadly accepted, definitive list of conditions used as indicators of poor housing/environmental health in Australia. Refining a widely accepted list of housing-associated ID outcomes could be useful to streamline evaluation and monitoring within research, service provider and policy settings.

A more granular understanding of ID-HLP associations could help to inform targeted investment in specific HLPs with the greatest health benefits. Defined in an Australian Indigenous community context, the HLPs provide a useful, holistic and meaningful framework to evaluate the functional state of housing and health infrastructure. Nonetheless, few studies included in the review used the HLP framework to describe housing exposures. Similarly, each study used a unique mix of health data sources and terminology to describe ID outcome measures. Studies were therefore not comparable spatially, temporally or methodologically and could only be synthesised by retrospectively classifying exposures and outcomes according to common classification systems (in this case, the HLPs and ICPC-2 body systems, respectively). We recommend that further research in this field involve prospective classification of housing exposures by the HLPs *and* validation of ID outcomes for meaningful data interpretation and consistency moving forward.

Housing improvements and capacity to monitor health impacts represent major priorities for Indigenous communities and peak organisations.^2459^,^61^ In addition to signalling the need for increased funding and structural changes, the *NACCHO Policy Position Paper: Aboriginal Housing for Aboriginal Health* makes an urgent call to ‘Implement a rigorous national research, evaluation and data collection programme that monitors the impact of Aboriginal and Torres Strait Islander housing policy against health indicators’.^24^ The need for such evaluation and monitoring capacity is also highlighted in the *National Agreement on Closing the Gap – Priority Reform Four: Shared Access to Data and Information at a Regional Level*^62^ and echoed in a range of current Indigenous housing and environmental health plans.^63^,^65^ Academic studies are unsuitable for this purpose because they rely on intensive data collection, ad hoc funding and third-party (usually non-Indigenous) organisations with limited resources for translation; they provide snapshots but are usually not repeated to determine temporal trends, and they have not always upheld Indigenous data sovereignty principles. Moving beyond siloed epidemiological studies, research attention could be refocused on the co-development of robust monitoring tools to strengthen capacity for sovereign data collection within Indigenous community-controlled health and housing organisations.^23^

### Limitations

The impacts of inadequate housing/HLP capacity on social and emotional wellbeing and chronic disease were not captured by this review. We nonetheless acknowledge the inter-related and far-reaching impacts of housing on physical, social and emotional health and wellbeing and understand the challenges associated with applying an exclusively biomedical lens to this work. Current approaches to assessing and communicating the need for improvements in Indigenous housing lack sophistication and effectiveness. However, housing is a human right and the inability to define or measure the impact of the various elements of inadequate housing should not hinder progress in improving housing condition.^66 67^

Most studies were descriptive and did not actually assess causality or control for the many confounding factors which can influence ID outcomes, despite our use of the phrase ‘ID-HLP associations tested’ as an aggregate term. Specific ID-HLP associations reported therefore remain speculative. Some ID outcomes may have been understudied or missed by this review, and some may be reported less frequently but have a more significant impact and cost, for example, ARF and RHD. The focus on identified ID outcomes and the strength of their association with housing is inherently tied to research priorities, study design, statistical power and methodological quality. Nevertheless, the present focus on ID outcomes is considered useful for discussion and concept development. The cultural and professional diversity of our authorship team, leadership by the IGC, methodological rigour, and alignment to meaningful classification frameworks for understanding the impacts of housing on health are strengths of this work.

## Conclusion

For decades, research has sought to describe the connection between inadequate housing and ID outcomes in Australia, particularly for Indigenous children and families. Access to safe, reliable water (HLP1-3) and sufficient housing stock to reduce the negative effects of crowding (HLP5) are frequently tested and positively associated with ID outcomes affecting the skin, respiratory and digestive systems. However, high-quality evidence remains piecemeal, and heterogeneity between methodologies limits the synthesis and actionability of this work. Agreed measurement approaches and validated data collection tools are needed to enable longitudinal monitoring and may be better positioned to inform decision making. We encourage the exploration of routinely collected primary care data and metrics that are consistent with Indigenous Data Sovereignty principles.

## Supplementary material

10.1136/bmjph-2025-003531 online supplemental file 1 1, 2

## Data Availability

All data relevant to the study are included in the article or uploaded as supplementary information.
