# Supplementary material for: Infectious disease outcomes associated with inadequate housing and access to healthy living practices in Australia: a systematic review
Source: BMJ Public Health. 2026 Feb 27;4(1):e003531. doi: 10.1136/bmjph-2025-003531 (PMC12959062; doi:10.1136/bmjph-2025-003531)
Supplement: online supplemental file 1 [file bmjph-4-1-s001.pdf]

Infectious disease outcomes associated with inadequate housing and access to Healthy Living Practices in Australia: A systematic review

---

SUPPLEMENTAL MATERIAL

Consider Statement

Table S1. The research addressed each item of the CONSIDER Statement.

**1. GOVERNANCE**	
Describe partnership agreements between the research institution and Indigenous-governing organization for the research, (e.g., Informal agreements through to MOU (Memorandum of Understanding) or MOA (Memorandum of Agreement).	• This research is underpinned by Standards for the Conduct of Aboriginal Health Research (The Kids Research Institute of Australia) and standards of associated organisations that are consistent with best practice advice from the NHMRC (https://www.nhmrc.gov.au/research-policy/ethics/ethical-guidelines-research-aboriginal-and-torres-strait-islander-peoples).<br>• It forms part of the NHMRC-funded Stopping Acute Rheumatic Fever to Strength Health (STARFISH) program of work. The hallmark of STARFISH is the agenda-setting and leadership by an Indigenous Governance Council (IGC). The rationale, methodology, aims and implications of this research were co-developed by lead investigators and the IGC. These mechanisms emphasise the importance of Indigenous governance and ensure accountability and harm minimisation.<br>• RB and VW are members of the IGC and have contributed to all phases of this research on a day-to-day level.<br>• The research supports Aboriginal and Torres Strait Islander self-determination, self-governance and data sovereignty through development of a monitoring tool.<br>• Study-specific data, community names and locations have not been reported or discussed in the manuscript.
Describe accountability and review mechanisms within the partnership agreement that addresses harm minimization.	
Specify how the research partnership agreement includes protection of Indigenous intellectual property and knowledge arising from the research, including financial and intellectual benefits generated (e.g., development of traditional medicines for commercial purposes or supporting the Indigenous community to develop commercialization proposals generated from the research).	
**2. PRIORITISATION**	
Explain how the research aims emerged from priorities identified by Indigenous stakeholders, governing bodies, funders, non-government organization(s), stakeholders, consumers, and empirical evidence.	• The rationale for the review and concept of the monitoring tool emerged from priorities outlined by NACCHO, as well as Closing the Gap Priority Reforms, and Indigenous Data Sovereignty principles.<br>• The review forms part of a broader stepwise body of work including a perspective article, Key Knowledge Holder interviews, consensus-based decision-making workshops, and partnerships with remote community-controlled health organisations for validation studies. This will ensure the priorities of all stakeholders and end-users are reflected in the work and that the tool will be of relevance and benefit.
**3. RELATIONSHIPS**	
Specify measures that adhere and honour Indigenous ethical guidelines, processes, and approvals for all relevant Indigenous stakeholders, recognizing that multiple Indigenous partners may be involved, e.g., Indigenous ethics committee approval, regional/national ethics approval processes.	• Ongoing collaboration with the IGC, as well as informal discussions with Indigenous colleagues, community members, students and health care providers demonstrate the commitment of the research team to relationships and respect for Indigenous stakeholders, culture and histories.<br>• Aboriginal and Torres Strait Islander people have and continue to be involved in all phases of the research process (design, funding, implementation, analysis, dissemination).<br>• We seek to foster constructive discussion and collaborations between stakeholders in what is currently a politicised space.<br>• Ethics approval was not required to produce this article.
Report how Indigenous stakeholders were involved in the research processes (i.e., research design, funding, implementation, analysis, dissemination/recruitment).	
Describe the expertise of the research team in Indigenous health and research.	• RB is a Wonnarua woman currently residing in Kununurra. She is an experienced social scientist and senior research fellow, specialising in culturally safe health promotion, and is the Indigenous lead of the END RHD Program at The Kids.<br>• VW is a senior Noongar woman with over 40 years of experience in health at state and national levels. She is a cardiac nurse and sits on a range of boards, committees and research projects. She is well known and respected for her work, particularly in relation to Indigenous heart health and Closing the Gap.<br>• RW is a non-Indigenous general practitioner experienced in research and clinical practice in remote Indigenous community settings. She is the lead author of the RHD Endgame Strategy and a senior research fellow with The Kids and *Yardhura Walani* at the Australian National University.<br>• JK is a non-Indigenous epidemiologist with a career in applying linked data methods to the study of heart disease among Indigenous people with a view to build capacity and advocate for improvements in environmental health.<br>• AB is a non-Indigenous paediatric infectious disease specialist at Perth Children's Hospital and leads a large research portfolio at The Kids. Her work is focused on infectious diseases research and clinical trials with

	significance to skin health and ARF/RHD prevention for Aboriginal and Torres Strait Islander children.<br>• KS, KA, IS, and SS are non-Indigenous early career researchers with relevant training in biomedical science and Aboriginal and Torres Strait Islander health research.
**4. METHODOLOGIES**	
Describe the methodological approach of the research including a rationale of methods used and implication for Indigenous stakeholders, e.g., privacy and confidentiality (individual and collective)	• ID outcomes and housing exposures were codified using existing classification systems (the HLPs and ICPC-2 body system classes, respectively) chosen for their relevance to Indigenous communities and most applicable to the development of a monitoring tool for use by community-controlled health organisations.<br>• Both quantitative and qualitative study designs were considered to capture experiences and perspectives that may be overlooked in quantitative epidemiological studies.<br>• We acknowledge that the impacts of colonisation, racism and social injustice are deep and ongoing. It is of utmost importance that research does not continue to contribute to these impacts. Acknowledgement of these realities underpins the concept of the monitoring tool recommended by way of this review.
Describe how the research methodology incorporated consideration of the physical, social, economic and cultural environment of the participants and prospective participants. (e.g., impacts of colonization, racism, and social justice). As well as Indigenous worldviews.	
**5. PARTICIPATION**	
Specify how individual and collective consent was sought to conduct future analysis on collected samples and data (e.g., additional secondary analyses; third parties accessing samples (genetic, tissue, blood) for further analyses).	• There was no primary data collection or research participants involved in this review.<br>• The recommendation to develop a monitoring tool will necessitate ongoing research and collaboration. We acknowledge that this should not be burdensome to individuals, communities or organisations. The data ecosystem will be controlled by Indigenous researchers and stakeholders as far as possible, with ample support from non-Indigenous collaborators to manage the workload. RB and VW, along with the IGC, Key Knowledge Holders, Indigenous collaborators and community partners will continue to inform and guide this work.
Described how the resource demands (current and future) placed on Indigenous participants and communities involved in the research were identified and agreed upon including any resourcing for participation, knowledge, and expertise.	
Specify how biological tissue and other samples including data were stored, explaining the processes of removal from traditional lands, if done, and of disposal.	
**6. CAPACITY**	
Explain how the research supported the development and maintenance of Indigenous research capacity (e.g., specific funding of Indigenous researchers).	• This review will continue to inspire discussion and advance the evidence base and rationale for development of a monitoring tool.<br>• It will support new and ongoing opportunities for Aboriginal and Torres Strait Islander students and established researchers as the work progresses.<br>• All co-authors seek regular professional development opportunities in Indigenous health, research standards and community engagement.<br>• Our ways of working result in two-ways knowledge sharing and benefits for both Indigenous and non-Indigenous authors and collaborators.
Discuss how the research team undertook professional development opportunities to develop the capacity to partner with Indigenous stakeholders?	
**7. ANALYSIS AND INTERPRETATION**	
Specify how the research analysis and reporting supported critical inquiry and a strength-based approach that was inclusive of Indigenous values.	• Concerted effort was made to avoid the use of deficit language surrounding the health and infectious disease burden of inadequate housing, rather focusing on framing as strengths-based capacity building for the HLPs.<br>• RB and VW contributed to the interpretation and reporting of all data, including the Aboriginal and Torres Strait Islander Quality Appraisal tool scores, and provided specific review of wording throughout.
**8. DISSEMINATION**	
Describe the dissemination of the research findings to relevant Indigenous governing bodies and peoples.	• End users of this work are wide ranging and expected to include Aboriginal and Torres Strait Islander community-controlled health organisations.<br>• We will exchange findings of this review and companion pieces with important stakeholders, governing bodies, partner organisations, and Key Knowledge Holders, at planned workshops and conferences, and in regular working group meetings over the coming years.<br>• The manuscript highlights the need for monitoring and evaluation to inform evidence-based policy, investment and decision making by community-controlled organisations. It discourages further epidemiological studies and makes strong recommendations to translate knowledge into action.
Discuss the process for knowledge translation and implementation to support Indigenous advancement (e.g., research capacity, policy, investment).	

Search terms

Table S2. Search terms used to search scientific databases PubMed, Scopus, and Informit as of May, 2024.

**i)**	hous* OR home OR dwelling OR accommodation OR “living space” OR “living quarters” OR “healthy living practices”
	AND
**ii)**	infection* OR “communicable disease” OR bacterial OR viral OR virus OR parasit* OR fungal OR disease transmiss* OR “contagious disease”
	AND
**iii)**	Australia* OR (Aborigin* OR Torres Strait Island*)

Table S3. Terms used in Ovid MEDLINE.

#	Query	Results (May 2024)
1	*housing/ or hous*/ or home/ or dwelling/ or accomodation/ or living space.mp. [mp=title, book title, abstract, original title, name of substance word, subject heading word, floating sub-heading word, keyword heading word, organism supplementary concept word, protocol supplementary concept word, rare disease supplementary concept word, unique identifier, synonyms, population supplementary concept word, anatomy supplementary concept word]	10,368
2	Housing.mp.	63,403
3	Infections.mp.	1,542,776
4	exp Communicable diseases/ or exp bacterial infection/ or exp fungal infection/ or exp virus infection/ or exp parasitosis/ or exp transmi* disease/ or exp infectious disease/ or exp contagious disease/	2,328,295
5	Health Services, Indigenous/st [Standards]	404
6	Hygiene.mp.	93,889
7	Infections/th [Therapy]	2,524
8	(Epidemiology or Prevalence or Recurrence or Risk Factors).mp. [mp=title, book title, abstract, original title, name of substance word, subject heading word, floating sub-heading word, keyword heading word, organism supplementary concept word, protocol supplementary concept word, rare disease supplementary concept word, unique identifier, synonyms, population supplementary concept word, anatomy supplementary concept word]	3,950,439
9	*Urban Health/sn [Statistics & Numerical Data]	1,501
10	(exp "Native Hawaiian or Other Pacific Islander"/ or aborig*.tw. or indigenous.tw.) and (australia*.tw. or exp australia/)	12,673
11	Indigenous Australians/ or Aboriginal.mp. [mp=title, book title, abstract, original title, name of substance word, subject heading word, floating sub-heading word, keyword heading word, organism supplementary concept word, protocol supplementary concept word, rare disease supplementary concept word, unique identifier, synonyms, population supplementary concept word, anatomy supplementary concept word]	10,722
12	1 or 2	63,863
13	3 or 4 or 5 or 6 or 7 or 8 or 9	5,981,351
14	10 or 11	16,454
15	12 and 13 and 14	186

Housing-related exposures

Healthy Living Practices- housing-related exposures defined by community

In 1987, the Uwankara Palyanku Kanyintjaku (UPK) Report[1] articulated nine Healthy Living Practices (HLPs) which pertain to specific elements of the home environment that enable people to live healthy lives. The HLPs span fundamental human rights to adequate housing, water and sanitation[2] which have strong pathways to the prevention of infectious diseases (Table S4). They are also relevant to non-communicable disease and wellbeing more broadly, although causal relationships are more difficult to demonstrate.[3] Defined in an Australian Indigenous community context, the HLPs provide a useful, holistic and meaningful framework to evaluate the state of housing and health infrastructure and ensure accountability in the public housing sector; [4] they also account for the overlap in services provided by the individual household and wider community. Whilst the lens of the HLPs and of our work is focused on Aboriginal and Torres Strait Islander communities, the need and right to access adequate housing for good health pertains to all people. The HLPs and the work in this review are therefore broadly relevant.

For consistency, explanatory housing/HLP variables reported in each study included in the review were classified by the authors (KS and KA) according to the nine respective HLP categories and a tenth category (HLP 10) was created for general housing problems. For example, "shower not working" was classified as *HLP1 (washing people)*; "toilet infrastructure problems" was classified as *HLP3 (removing wastewater safely)*; "household size", "bedroom sharing" or "overcrowded" was classified as *HLP5 (reducing the negative impacts of crowding)*; "major structural problems", "housing problems" or collective housing-related issues were classified as *HLP10 (general housing problems)*. Access to safe drinking water, including the safety of household rainwater tanks, was classified as *HLP3 (removing wastewater safely)* in the absence of a HLP category that incorporates this specifically. Original exposures and corresponding HLP classifications are provided in Supplementary material table S5.

Occasionally, measured variables related to more than one HLP category e.g., "major plumbing problems" may have been measured once in a given study but classified as *HLP1 (washing people), HLP2 (washing clothes and bedding)*, and *HLP3 (removing wastewater safely)* in our data extraction. Conversely, some variables were measured in multiple ways but related to only one HLP category e.g., "food storage", "food transport" and "handling meat" may have represented three measurements in a given study but only one classification, *HLP4 (ability to store, prepare and cook food)*, was used in our data extraction. The effect of each tested ID-HLP association was coded as 0- not measured, 1- measured and positive association found, 2- measured and no association found, or 3- measured and negative association found.

Table S4. Healthy Living Practices (HLPs), adapted from the UPK Report.[1] Functional health hardware is described by Pholeros et al.[5] Relationships to infectious disease are based on current scientific and public health understanding, supported by selected references and studies included in this review.

Healthy Living Practices			Functional health hardware	Relationship to infectious disease
1	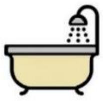	**Washing people**	Wet area, hot and cold-water supply, soap, a shower/bath, handbasin, working drainage, privacy	Washing hands and bodies with soap and water removes pathogens and prevents infections, particularly among children.[6,7] Community swimming pools may be an adjunct to this HLP.[8]
2	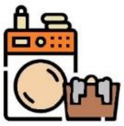	**Washing clothes and bedding**	Washing machine/community laundry, (hot) water supply, electricity, detergent, drainage, drying area	Washing clothes and bedding kills/removes pathogens that persist on fabrics and can be transmitted via fabric fomites.[7,9] Community laundries may be an adjunct to this HLP.[10]
3	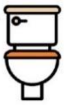	**Removing wastewater safely**	Toilet, drainage and vent pipes, household or community-level wastewater treatment	Untreated waste and wastewater contain abundant pathogens and pollutants.[7,11] Includes access to safe drinking water.
4	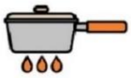	**Improving nutrition; the ability to store, prepare and cook food**	Stovetop and oven, refrigerator and freezer, electricity supply, kitchen bench and splashback, storage cupboards, cookware	Some pathogens can be transmitted via food that has not been stored or cooked properly.[12] Diet influences many health outcomes and plays a role in immunity and recovery from infections.[13]
5	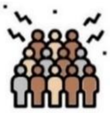	**Reducing the negative impacts of crowding**	Sufficient volume of houses, ensuring access to functional health hardware among houses, spaces to accommodate guests	Household crowding can overwhelm health hardware (e.g., toilets and showers) and is a major contributor to the spread of infectious diseases.[14-16] This HLP seeks to alleviate the negative impacts of household crowding that can co-exist with the positive social and cultural benefits of being together.
6	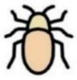	**Reducing the negative effects of animals, insects and vermin**	Fencing, insect screens, pest control, drainage and ventilation	A range of infectious diseases can be transmitted via contact with insects (e.g., mosquitoes), pets (e.g., cats and dogs), and vermin (e.g., mice and rats).[17-20]
7	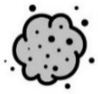	**Reducing the health impacts of dust**	Appropriate building finishes, taps and irrigation, established vegetation, fencing	Dust can irritate eyes, skin, and mucus-secreting parts of the body. Adhesion and transmission of respiratory pathogens is enhanced by airborne dust.[21] Dust also reduces the lifespan of other health hardware (e.g., washing machines).
8	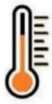	**Controlling the temperature of the living environment**	Insulation and ventilation, functioning windows, established vegetation, appropriate air conditioning systems	The survival and transmission of pathogens may be different in hot temperatures, wet, dry or humid conditions, or cold weather when people need to sleep close together for warmth. Climate change is likely to exacerbate some infectious diseases, especially neglected tropical diseases.[22]
9	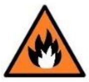	**Reducing hazards that cause trauma**	Regulated hot water temperature, stoves and heaters, non-slip surfaces, security, tidy yards and community spaces	Safe houses and yards prevent unintentional injuries; minor skin trauma, e.g., burns and cuts, can become infected with pathogens from the surrounding skin or source of the injury.[23]

Infectious disease (ID) outcomes

International Classification of Primary Care (ICPC-2) body system classes

The International Classification of Primary Care, 2nd edition (ICPC-2) is the most widely used international clinical coding system that allows primary care providers to record symptoms and diagnoses at the point of care.[24] The ICPC-2 is divided into 17 chapters by body systems representing the localisation of the problem or disease (Table S5).

During data extraction, ID outcomes were recorded as described then classified according to one of the ICPC-2 body system classes. For example, “otitis media” and “middle ear pain” were classified as *ear*; “pneumonia”, “upper respiratory tract infection” and “sore throat” were classified as *respiratory*; “impetigo”, “scabies” and “skin sores” were classified as *skin*. Outcomes describing ID symptoms or post-infectious sequelae were also included (e.g., hearing and eyesight issues, ARF/RHD, acute glomerulonephritis) and classified by ICPC-2 body system classes.

Terminology used to describe the 171 ID outcomes varied and not all were unique conditions. For example, skin sores were described as “skin sores”, “skin infection”, “dermal infection”, “skin/soft tissue infection”, “infected skin sores”, “pyoderma”, “non-scabies pyoderma”, “bacterial skin infection”, “skin lesions”, “scabies with or without skin infection”, or “impetigo”. Some diagnoses included additional information, e.g., “ear infection”, “otitis media”, “chronic suppurative otitis media”, “otitis media with or without perforation”, “tympanic membrane perforation following middle ear infection”. There were also different levels of specificity in reported diagnoses e.g., “mosquito-borne disease” vs. “dengue fever”; “respiratory infection” vs. “pneumonia”. Some ID outcomes were symptoms, rather than confirmed diagnoses e.g., “sore throat”, “wet/dry cough”, “vomiting”.

Table S5. International Classification of Primary Care (ICPC-2) body system classes and examples of associated infectious diseases.

ICPC2 Body system		Infections ONLY
A	General / unspecified	Tuberculosis<br>Measles<br>Chickenpox<br>Malaria<br>Rubella<br>Infectious mononucleosis<br>Viral exanthem other<br>Viral disease other/NOS<br>Infectious disease other/NOS<br>Bone and joint infection<br>Dengue fever<br>Ross River Fever<br>Meningitis
B	Blood, blood forming organs and immune mechanisms	Lymphadenitis acute<br>Lymphadenitis non-specific<br>Bloodstream infection

D	Digestive	Gastrointestinal infection<br>Mumps<br>Viral hepatitis<br>Gastroenteritis presumed infection<br>Giardiasis<br>Diarrhoea/vomiting<br>Salmonella infection<br>Parasitic infection
F	Eye	Conjunctivitis- infectious<br>Conjunctivitis- allergic<br>Blepharitis/stye/chalazion<br>Eye infection/inflammation other<br>Trachoma
H	Ear	Otitis externa<br>Acute otitis media/myringitis<br>Eustachian salpingitis<br>Chronic otitis media<br>Suppurative otitis media
K	Cardiovascular	Infection of circulatory system<br>Rheumatic fever/heart disease
L	Musculoskeletal	Infections musculoskeletal system
N	Neurological	Poliomyelitis<br>Meningitis/encephalitis<br>Tetanus<br>Neurological infection other
P	Psychological	None
R	Respiratory	Whooping cough<br>Strep throat<br>Pharyngitis<br>Upper respiratory infection acute<br>Sinusitis acute/chronic<br>Tonsillitis acute<br>Laryngitis/tracheitis acute<br>Acute bronchitis/bronchiolitis<br>Chronic bronchitis<br>Influenza<br>COVID-19<br>Pneumonia<br>Pleurisy/pleural effusion<br>Respiratory infection other
S	Skin	Warts<br>Infected finger/toe<br>Boil/carbuncle<br>Skin infection post traumatic<br>Herpes zoster<br>Herpes simplex<br>Scabies/other acariasis<br>Pediculosis/skin infection other<br>Dermatophytosis<br>Moniliasis/candidiasis skin<br>Skin infection other<br>Impetigo / pyoderma<br>Tinea<br>Molluscum contagiosum
T	Endocrine/Metabolic and Nutritional	Endocrine infection
U	Urological	Pyelonephritis/pyelitis<br>Cystitis/urinary tract infection other<br>Urethritis<br>Glomerulonephritis
W	Pregnancy, Childbearing, Family Planning	Puerperal infection/sepsis<br>Infection complicating pregnancy
X	Female Genital	Syphilis female<br>Gonorrhoea female<br>Genital candidiasis female<br>Genital trichomoniasis female<br>Pelvic inflammatory disease<br>Genital herpes female<br>Condylomata acuminata female<br>Chlamydia infection genital (f)

Y	Male Genital	Syphilis male<br>Gonorrhoea male<br>Genital herpes male<br>Prostatitis/seminal vesiculitis<br>Orchitis/epididymitis<br>Balanitis<br>Condylomata acuminata male
Z	Social Problems	None

Exclusion criteria

The causal pathways between homelessness and ID outcomes are multi-factorial and studies investigating the association were excluded. Studies investigating health associated with different housing arrangements (e.g., rental, social housing, home ownership) and institutions (e.g., prisons, early education centres and aged care facilities) were also excluded (non-household study setting). Sexually transmitted IDs and IDs arising from sharing drug paraphernalia were not considered directly related to housing for the purpose of this review. Studies were excluded if ID outcomes/transmission to humans were not measured, even if relevant housing conditions (e.g., functional state of health hardware) or pathogens (e.g., mould, intestinal parasites in pets) were measured (no ID outcome measure). Similarly, studies that investigated ID outcomes were excluded if housing-related exposures were not measured, even if housing was discussed as a potential risk factor and/or solution (no HLP exposure measure). Systematic and narrative reviews based on secondary data were excluded (review based on secondary data), but all reviews were checked for additional references that appeared relevant by title, which were added to Covidence and screened (in-text citation searching).

Quality appraisal methods

The methodological quality of each study was determined using Joanna Briggs Institute critical appraisal tools (JBI tools) appropriate to each study design (https://jbi.global/critical-appraisal-tools). Two authors (KA-100%, KS-35%) independently scored studies against JBI criteria for study design, conduct and analysis. Guidance was provided and any conflicting scores were resolved by experts in epidemiology (JK, IS and SS). Mixed methods studies and models were not scored for biomedical quality in the absence of relevant JBI tools. In parallel, quality assessment of applicable studies was conducted using the Aboriginal and Torres Strait Islander Quality Appraisal Tool (QAT) [83], which enables an assessment of study quality and value with respect to the cultural appropriateness of research involving Aboriginal and Torres Strait Islander people, families and communities. As per the JBI tools, the Aboriginal and Torres Strait Islander QAT was independently applied to relevant studies by two authors (KA-100%, KS-35%). Guidance was provided and any conflicting scores were resolved by Aboriginal co-authors (VW, RB). For both

the JBI tools and Aboriginal and Torres Strait Islander QAT, scores were recorded as 1 for 'yes' or 'partially', or 0 for 'no', 'unclear' or 'not applicable'. Scores for each study were totalled then normalised as percentages. Raw scores are supplied in Tables S7 and S8. No studies were to be excluded on the basis of quality.

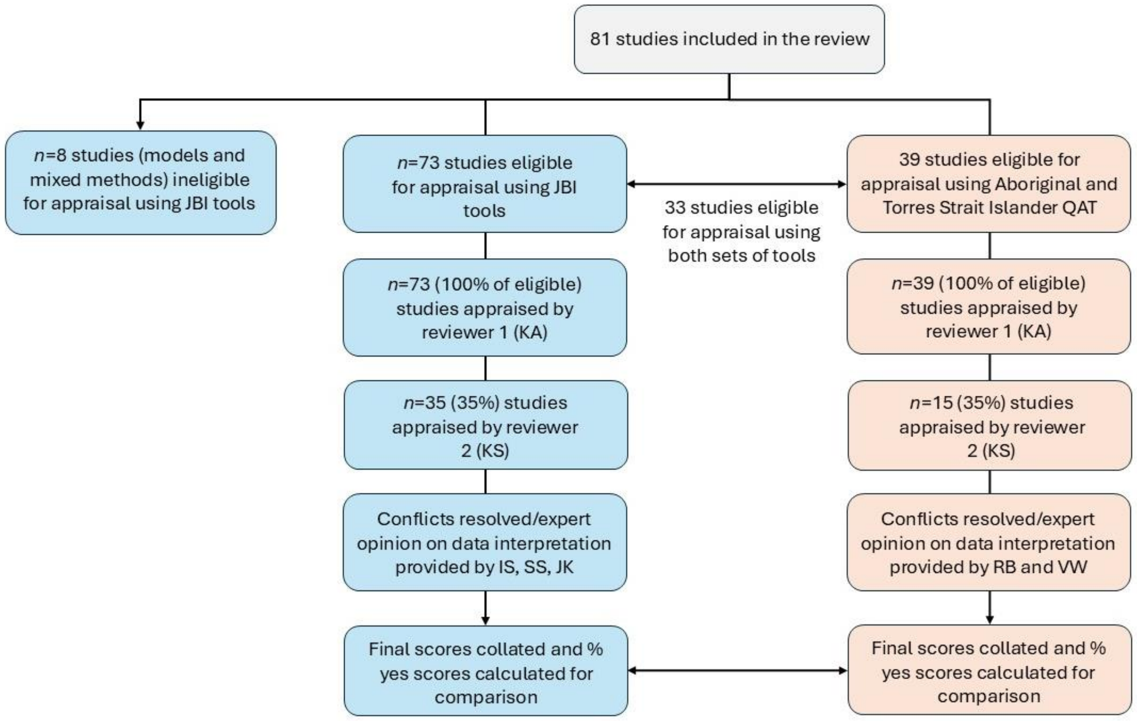

Figure S1. Process used to assess the methodological quality of articles included in the review from both biomedical and Indigenous perspectives using Joanna Briggs Institute (JBI) tools and the Aboriginal and Torres Strait Islander Quality Appraisal Tool (QAT), respectively.

Data summary

Table S6. Summary of data from 81 reviewed studies that measured infectious disease (ID) outcomes associated with inadequate housing. ID outcomes were classified according to ICPC-2 body system classes and housing exposures were classified by the Healthy Living Practices (HLPs). MMM: Modified Monash Model classification. NSW: New South Wales, Qld: Queensland, SA: South Australia, Vic: Victoria, WA: Western Australia.

First author (publication date)	Type of study	Location / Setting (Jurisdiction) MMM	Population	Age category	Infectious disease (ID) outcomes			Exposures		General results	Ref
					ID as reported	Relevant ICPC2 body system	ID data source	As reported	HLP categories		
Akter (2017)	Cross-sectional	National. MMM mixed	Mixed	Mixed	i. Dengue fever	i. General	Notifiable disease registry	Rainwater tanks, household structure	3	Incidence and distribution of dengue related to socio-demographic factors such as households having rainwater tanks, overseas arrivals, Indigenous populations and terrace houses.	2
Andersen (2016)	Qualitative	Western Sydney (NSW). MMM 1	Aboriginal or Torres Strait Islander	Mixed	i. Cold and flu<br>ii. Gastroenteritis<br>iii. Ear infections<br>iv. Chest infections<br>v. Skin infections	i. Respiratory<br>ii. Digestive<br>iii. Ear<br>iv. Respiratory<br>v. Skin	Self-reported	Participants believed a key driver for high rates of communicable diseases in their community was overcrowding.	5	Many of the specific health concerns participants attributed to poor housing echo existing epidemiological research findings.	3
Andersen (2018)	Cohort	Mt Druitt, Campbelltown, Wagga Wagga, Newcastle (NSW)<br>MMM 3	Aboriginal or Torres Strait Islander	Child/ adolescent (0-17 y)	i. Gastrointestinal infection (recurrent)	i. Digestive	Self-reported	Major plumbing, electrical or structural housing problems, damp or mildew, vermin, crowding, and ability to make the home warm enough in winter.	1, 2, 3, 5, 6, 8, 10	Gastrointestinal infection related to major structural problems, major plumbing problems, damp or mildew, feeling crowded.	4
Bailie (2012)	Cohort	10 communities (NT)<br>MMM mixed (although most remote)	Aboriginal or Torres Strait Islander	Child (0-7 y)	i. Scabies with or without skin infection (includes boils)<br>ii. Gastroenteritis<br>iii. Ear infection<br>iv. Respiratory infection<br>v. Skin infection (includes boils) with no scabies	i. Skin<br>ii. Digestive<br>iii. Ear<br>iv. Respiratory<br>v. Skin	Self-reported	Infrastructure surveys involved an inspection/ testing of the functional state of each house in relation to the infrastructure required to conduct HLPs	1, 2, 3, 4, 5, 6, 7, 8, 9, 10	Improvements in certain HLPs and overall infrastructure function resulted in positive change in reports of illness. E.g., washing clothes and bedding, removing human waste, separating humans and animals and reducing crowding were associated with positive change in reports of skin infections.	5
Bailie (2005)	Cross-sectional	Three communities typifying	Aboriginal or Torres Strait Islander	Child (0-7 y)	i. Skin infections (scabies and/or bacterial infection)	i. Skin	Primary care	A survey of dwelling condition and availability of cleaning items; the	1, 2, 3, 5, 10	Variables with the strongest and most consistent association with incidence	6

First author (publication date)	Type of study	Location / Setting (Jurisdiction) MMM	Population	Age category	Infectious disease (ID) outcomes			Exposures		General results	Ref
					ID as reported	Relevant ICPC2 body system	ID data source	As reported	HLP categories		
		conditions prevalent in remote Indigenous communities in Australia (NT) MMM 6-7						functional state of each item was scored by an environmental health/housing officer using standardised survey forms used for housing management purposes (relating to HLPs)		of skin infections are those reflecting household composition and social process. Of the three HLP variables, having the facilities to remove faeces appears most important; the age of the dwelling is a modifying factor.	
Bailie (2010)	Cross-sectional	10 communities (NT) MMM mixed (although most remote)	Aboriginal or Torres Strait Islander	Child (0-7 y)	i. Respiratory infection<br>ii. Diarrhoea and/or vomiting<br>iii. Ear infection<br>iv. Scabies with or without skin infection (includes boils)<br>v. Skin infection (includes boils) with no scabies	i. Respiratory<br>ii. Digestive<br>iii. Ear<br>v. Skin<br>vi. Skin	Self-reported	A systematic detailed survey of the functional state of household infrastructure relating to HLPs	1, 2, 3, 4, 5, 6, 7, 8, 9, 10	For unadjusted associations with primary variables, significant associations between each of the child illnesses and poor infrastructure were found for: scabies and removal of rubbish and control of dust; diarrhoea and/or vomiting and preparation and storage of food; ear infection and toilet infrastructure and poor infrastructure overall.	7
Boreham (1986)	Cohort	Mount Isa (Qld) MMM 3	Not specified	NA	i. Diarrhoea or giardiasis (proven or suspected)	i. Digestive	Mixed sources (self-reported and researcher collected)	Questionnaire and census data relating to household water supply, sewage disposal, swimming, and household cats or dogs	3, 6	No evidence that people were acquiring the parasite from water or pets	8
Brown (2015)	Cohort	Melbourne (Vic) MMM 1	Not specified	Mixed	i. Influenza-like illness	i. Respiratory	Mixed sources (self-reported and researcher collected)	Household size and number of children in the house	5	There was no association with household size; the strongest predictor of transmission was the age group of the index case	9
Carcione (2011)	Cross-sectional	(WA) MMM mixed	Mixed	Mixed	i. Pandemic influenza A (H1N1)2009	i. Respiratory	Notifiable disease registries	Household size and bed sharing	5	Strongest predictor was age	10
Carver (2008)	Cross-sectional	Mallee region-including numerous rural towns (Vic) MMM 5	Not specified	Not specified	i. Ross River virus	i. General	Researcher collected	House mouse abundance	6	Mice as short-lived highly fecund hosts may have a profound influence on disease transmission	11
Chakraborty (2021)	Qualitative	123 remote communities (NT) MMM 6-7	Aboriginal or Torres Strait Islander (priority)	Mixed	i. Skin infection<br>ii. Ear infection<br>iii. Respiratory infection	i. Skin<br>ii. Ear<br>iii. Respiratory	Self-reported	Group concept mapping included indicator statements such as: access to power and water,	1, 2, 3, 9, 10	Essential services related to indicator statements were of highest importance for	12

First author (publication date)	Type of study	Location / Setting (Jurisdiction) MMM	Population	Age category	Infectious disease (ID) outcomes			Exposures		General results	Ref
					ID as reported	Relevant ICPC2 body system	ID data source	As reported	HLP categories		
								sewerage and septic system, maintenance of housing infrastructure, regular rubbish collection, and access to continuous water supply.		both chronic and infectious diseases	
Chen (2014)	Cross-sectional	National<br>MMM mixed	Mixed	Mixed	i. Acute respiratory infection	i. Respiratory	Self-reported	Environmental and demographic exposures (household size)	5	Significant difference in odds ratios for different sized households	13
Chisholm (2020)	Model	Reflective of a remote community<br>(NA)<br>MMM 6	Aboriginal or Torres Strait Islander	Mixed	i. Infection parameters were chosen to be consistent with influenza-like illness	i. Respiratory	Modelled	Number of houses, population size, mobility, number of core residents per dwelling	5	Outbreak intensity was lower when scenarios had lower household crowding and less fluid dwelling occupancy	14
Cooper (1986)	Cohort	Red Centre; Eastern Kimberley; Eastern Goldfields.<br>(WA)<br>MMM 7	Aboriginal or Torres Strait Islander	Child/ adolescent (0-14 y)	i. Acute follicular trachoma	i. Eye	Researcher collected	Water availability, showering, water/sewage conditions, and types of housing described for communities in each zone	1, 2, 3		15
Dossetor (2017)	Cohort	Fitzroy Valley<br>(WA)<br>MMM 7	Aboriginal or Torres Strait Islander	Child (7-9 y)	i. Infections of the gastrointestinal system<br>ii. Lower respiratory tract infections<br>iii. Upper respiratory tract infections<br>iv. Meningococcal meningitis<br>v. Mitral regurgitation secondary to rheumatic heart disease<br>vi. Post-infectious genitourinary complication (glomerulonephritis and tubule-interstitial nephritis)	i. Digestive<br>ii. Respiratory<br>iii. Respiratory<br>iv. General<br>v. Cardiovascular<br>vi. Urological	Hospital admission data	Number of residents per house, considered overcrowded by parents/caregivers, food insecurity	4, 5	A third of the cohort lived in households considered overcrowded by the parents/caregivers; 41.9% reported food insecurity. Food insecurity also reported more commonly in children who were hospitalised (48.3%) compared to those who were not (23.7%)	16
Edwards (1970)	Cohort	Walgett<br>(NSW)<br>MMM 6	Aboriginal or Torres Strait Islander	Child (0-4 y)	i. Ear infection<br>ii. Pneumonia<br>iii. Skin infection<br>iv. Gastroenteritis<br>v. Respiratory infection	i. Ear<br>ii. Respiratory<br>iii. Skin<br>iv. Digestive<br>v. Respiratory	Mixed sources (self-reported and researcher collected)	Examination of postnatal nutritional factors and a survey of the dwelling in which the children lived	1, 3, 4, 5, 10	Children were members of large families living under very poor conditions. Houses situated out of town were all substandard ranging from social housing to tin shacks with no electricity, sewage or running water, and an average of 8.9 persons per dwelling	17
Ewald (2003)	Cohort	A remote community 300	Aboriginal or Torres Strait Islander	Child/ adolescent (0-13 y)	i. Trachoma	ii. Eye	Researcher collected	Scoring using the NT Environmental Health Housing Survey, after	1, 2, 3, 4, 5, 10	The proportion of people living with "adequate" facilities improved.	18

First author (publication date)	Type of study	Location / Setting (Jurisdiction) MMM	Population	Age category	Infectious disease (ID) outcomes			Exposures		General results	Ref
					ID as reported	Relevant ICPC2 body system	ID data source	As reported	HLP categories		
		km from Alice Springs<br>(NT)<br>MMM 7						standardising their survey interpretation. Data analysed according to the HLPs. Works included new houses/renovations and sewage system.		Widespread inadequate housing and continued crowding, as well as uncertainty about compliance with antibiotic treatment, likely contributed to the lack of effect of this program	
Foster (2021)	Mixed method	National (focus on remote communities of NT)<br>MMM mixed	Aboriginal or Torres Strait Islander	Mixed	i. Shigellosis<br>ii. Helminthiases (including tapeworm, hookworm, strongyloidiasis, trichuriasis)<br>iii. Trachoma<br>iv. Acute rheumatic fever	i. Digestive<br>ii. Digestive<br>iii. Eye<br>iv. Cardiovascular	Mixed sources (linked data, notifiable disease registries)	Fourteen datasets were identified comprising relevant information on crowding, dwelling condition, health hardware, and maintenance/repair services	5, 10	Poor housing and lack of functioning health hardware contribute to poor health	19
Hall (2017)	Cohort	Caboolture (Qld)<br>MMM 1	Aboriginal or Torres Strait Islander (priority)	Child (< 5 y)	i. Acute respiratory illness with cough (ARIwC)	ii. Respiratory	Self-reported	Having mould in the house (questionnaire)	8	Having mould in the house was associated with recurrent ARIwC	20
Hanna (1996)	Cohort	Badu (NT)<br>MMM 7	Aboriginal or Torres Strait Islander	Adult	i. Japanese encephalitis	i. General	Researcher collected	Mosquito surveys and surveys of household characteristics that may have contributed to the outbreak, including presence of pigs and horses, and the state of repair of waste disposal (drainage and sewage systems)	3, 6	Pigs and pig pens kept within 50 m of the house, many surrounded by standing water; houses had defective waste disposal systems with wastewater or raw sewage overflowing into the house or yard, and septic tanks contained larvae	21
Harris (1990)	Cohort	Bourke (NSW)<br>MMM 6	Aboriginal or Torres Strait Islander	Child/ adolescent (0-14 y)	i. Pneumonia<br>ii. Respiratory diseases<br>iii. Scabies<br>iv. Trachoma<br>v. Tympanic membrane perforation (following middle ear infection)<br>vi. Staphylococcal skin infection<br>vii. Gastroenteritis	i. Respiratory<br>ii. Respiratory<br>iii. Skin<br>iv. Eye<br>v. Ear<br>vi. Skin<br>v. Digestive	Mixed sources (hospital admission data, researcher collected)	Survey of housing conditions; persons per bedroom, sewage system, water within 5 m of the house, a shower or bath, hot water, electricity, refrigeration, screens on windows	1, 3, 4, 5, 6, 10	There was a marked improvement in housing conditions between 1971-1984 and improvements in health	22
Harris (1984)	Cohort	Bourke (NSW)<br>MMM 6	Aboriginal or Torres Strait Islander (priority)	Child (0-3 y)	Lobar pneumonia	Respiratory	Hospital admission data	Number of persons living in the dwelling and whether the child lived in the reserve where housing was substandard	5, 10	Association with substandard housing	23
Hempenstall (2021)	Case control	Torres Strait Islands (Qld)<br>MMM 7	Aboriginal or Torres Strait Islander	Child/ adolescent (5-18 y)	i. Rheumatic heart disease<br>ii. Sore throat<br>iii. Skin sore	i. Cardiovascular<br>ii. Respiratory<br>iii. Skin	Researcher collected	Number of people living in households	5	The number of people living in households was not higher for children with RHD compared to controls	24

First author (publication date)	Type of study	Location / Setting (Jurisdiction) MMM	Population	Age category	Infectious disease (ID) outcomes			Exposures		General results	Ref
					ID as reported	Relevant ICPC2 body system	ID data source	As reported	HLP categories		
Heyworth (2003)	Cross-sectional	(SA)<br>MMM 3	Mixed	Child (4-5 y)	i. Gastroenteritis (vomiting and/or liquid bowel movements, or nausea and/or soft bowel movements combined with abdominal cramps)	i. Digestive	Self-reported	Diet, pets, contact with farm animals	4, 6	Unpasteurised dairy associated	25
Heyworth (2006)	Cohort	Rural SA or Adelaide Hills (SA)<br>MMM 3	Non-Indigenous Australian	Child (4-6 y)	i. Gastroenteritis/gastrointestinal symptoms	i. Digestive	Self-reported	Drinking untreated rainwater, rainwater tank construction and maintenance, contact with pets and farm animals	3, 6	No greater odds of gastroenteritis in children who drank rainwater compared to those who drank treated mains water	26
Hodgetts (2022)	Cross-sectional	Katherine region (NT)<br>MMM 6	Aboriginal or Torres Strait Islander	Mixed	i. Melioidosis (cutaneous)	i. General	Notifiable disease registries	Water samples collected from the river + patient data	2	Wading through floodwater and flood cleanup associated with condition	27
Hui (2021)	Model	Modeled based on dynamics of communities in remote Australia (NA)<br>MMM 6	Aboriginal or Torres Strait Islander	Mixed	i. COVID-19	i. Respiratory	Modelled	Household size and mixing	5	Interconnected and often overcrowded housing means that unmitigated scenarios result in high infection rates	28
Inglis (1999)	Cohort	A remote coastal community in tropical WA<br>MMM 6-7	Aboriginal or Torres Strait Islander	Adult	i. Melioidosis	i. General	Notifiable disease registries	Potable water supplies	3	Range of possible contributing factors affecting potable water supply	29
Jacoby (2011)	Cohort	Kalgoorlie-Boulder area (WA)<br>MMM 4	Mixed	Child (0-2 y)	i. Otitis media	i. Ear	Researcher collected	Number of children in the house, number of rooms in the house	5	Higher number of children in the house and lower number of rooms associated with increased risk	30
Kaminski (1977)	Cross-sectional	Maningrida (NT)<br>MMM 7	Aboriginal or Torres Strait Islander	Child/ adolescent (3-13 y)	i. *Trichophyton rubrum/Microsporum canis* infection (tinea capitis), scalp lesions or skin lesions (abdomen, thigh, leg)	i. Skin	Researcher collected	Exposure to dogs and cats	6	Same variant found in children, cats and dogs	31
Kerrigan (2021)	Qualitative	Three remote communities (NT)<br>MMM 7	Aboriginal or Torres Strait Islander	Mixed	i. Acute rheumatic fever/Rheumatic heart disease	i. Cardiovascular	Self-reported	Qualitative accounts of housing problems e.g., can smell sewage, need to address issues that lead to overcrowding, effectiveness of handwashing	1, 3, 5, 9, 10	Plumbing and water problems, and crowding were common.	32
La Vincente (2009)	Cohort	Two east Arnhem Land communities (NT)<br>MMM 7	Aboriginal or Torres Strait Islander	Mixed	i. Scabies<br>ii. Skin sores	i. Skin<br>ii. Skin	Mixed sources (self-reported and researcher collected)	Household crowding determined at household visits	5	ORs for acquiring scabies not associated with crowding, but similar between groups	33

First author (publication date)	Type of study	Location / Setting (Jurisdiction) MMM	Population	Age category	Infectious disease (ID) outcomes			Exposures		General results	Ref
					ID as reported	Relevant ICPC2 body system	ID data source	As reported	HLP categories		
Lansingh (2010)	Cohort	within the Anangu Pitjantjatjara Lands of Central Australia (SA) MMM 7	Aboriginal or Torres Strait Islander	Child/ adolescent (0-15 y)	i. Active trachoma	i. Eye	Researcher collected	Environmental health interventions directly related to HLPs; facial cleanliness, removing waste safely	1, 2, 3, 4, 5, 6, 7, 8, 9	Both communities achieved significant reductions in trachoma prevalence, but changes in environmental health were modest and could not be related. Few houses reached required scores in most HLP categories.	34
Leach (2016)	Cohort	25 Top End communities (NT) MMM 7	Aboriginal or Torres Strait Islander	Child (0-6 y)	i. Otitis media (acute with effusion/ without perforation/ with perforation/dry perforation/chronic suppurative otitis media)<br>ii. Scabies<br>iii. Tinea<br>iv. Skin sores or other skin condition<br>v. Nasal discharge (visible at 1 m distance)<br>vi. Cough (spontaneous or on request, wet or dry)	i. Ear<br>ii. Skin<br>iii. Skin<br>iv. Skin<br>v. Respiratory<br>vi. Respiratory	Researcher collected	Number of siblings, number of people and children living in the house, exposure to campfire smoke (?), whether the child washed with soap the previous day	1, 5	No difference between PCV vaccine groups; household factors similar between groups	35
Looker (2010)	Cross-sectional	(Vic) MMM mixed	Not specified	Mixed (child <18 y or adult)	i. Influenza-like illness (ILI), pH1N1, seasonal influenza, pandemic influenza	i. Respiratory	Primary care	Household details (number of children and adults in household)	5	Higher secondary attack rate with higher household numbers	36
Marshall (2011)	Cross-sectional	Brisbane (Qld) MMM 1	Not specified	Mixed	i. *Mycobacterium lentiflavum* infection	i. Respiratory	Notifiable disease registries	Potable water supply	3	Clinically significant illness associated with proximity to positive water site	37
Massey (2009)	Qualitative	Awabakal (Newcastle), Armajun (Inverell), Armidale, Biripi (Taree), Tamworth and Tobwabba (Forster) (NSW) MMM 3	Aboriginal or Torres Strait Islander	Adult	i. Influenza	i. Respiratory	Self-reported	Large social gatherings such as funerals and cultural celebrations, hand wash or hand gel supplies	1, 5		38
May (2016)	Cohort	National MMM mixed	Not specified	Mixed	i. Gastroenteritis	i. Digestive	Notifiable disease registries	Food storage and preparation in home and commercial settings resulting in outbreaks	4	More often associated with commercial settings than food preparation in the home. Primary factor was inadequate temperature control.	39
McBride (1998)	Cohort	Charters Towers (Qld)	Not specified	Adult (> 14 y)	i. Dengue	i. General	Mixed sources (self-reported and	House screening, presence of rainwater tanks on the property or within two	6		40

First author (publication date)	Type of study	Location / Setting (Jurisdiction) MMM	Population	Age category	Infectious disease (ID) outcomes			Exposures		General results	Ref
					ID as reported	Relevant ICPC2 body system	ID data source	As reported	HLP categories		
		MMM 4					researcher collected)	residential blocks, and the presence of evaporative cooling units			
McDonald (2009)	Mixed method	Remote community located in north-western Arnhem Land (NT)<br>MMM 7	Aboriginal or Torres Strait Islander	Child/ adolescent (<15 y)	i. Skin infection<br>ii. Diarrhoeal disease<br>iii. Respiratory disease	i. Skin<br>ii. Digestive<br>iii. Respiratory	Self-reported	Health hardware/infrastructure (toilets, washing machines, refrigerators); faeces or other decaying matter such as meat and other food remnants.<br>Health and hygiene knowledge survey (including regular bathing with soap).	1, 2, 3, 4, 5, 6, 10	Crowding, non-functioning essential housing infrastructure and poor standards of personal and domestic hygiene associated with high burden of infection.	41
McDonald (2010)	Mixed method	Central Arnhem Land<br>(NT)<br>MMM 7	Aboriginal or Torres Strait Islander	Child (< 7 y)	i. Respiratory infection<br>ii. Bronchiectasis<br>iii. Parasitic diseases<br>iv. Chronic suppurative otitis media<br>v. Scabies<br>vi. Group A streptococcal pyoderma<br>v. Post-streptococcal glomerulonephritis<br>vii. Rheumatic fever and rheumatic heart disease<br>viii. Trachoma	i. Respiratory<br>ii. Respiratory<br>iii. Digestive<br>iv. Ear<br>v. Skin<br>vi. Skin<br>vii. Urological<br>viii. Cardiovascular<br>ix. Eye	Mixed sources (self-reported and hospital admission data)	Household items considered necessary to carry out six HLPs: wash people, wash clothes, functioning toilet, remove wastewater, remove waste rubbish and prepare and store food + environmental contamination (faeces or decaying matter), average persons living in the house, general housing issues.	1, 2, 3, 4, 5, 9, 10		42
McDonald (2007)	Cohort	Central Australia, 80km from Alice Springs<br>(NT)<br>MMM 7	Aboriginal or Torres Strait Islander	Child/ adolescent (<15 y)	i. Pyoderma/skin sores<br>ii. Acute rheumatic fever/Rheumatic heart disease	i. Skin<br>ii. Cardiovascular	Mixed sources (self-reported and researcher collected)	Median number of people per bedroom/household size	5	High rates of crowding	43
McDonald (2008)	Cohort	3 remote communities in the Top End<br>(NT)<br>MMM 7	Aboriginal or Torres Strait Islander	Mixed	i. Pyoderma (skin sores, GAS isolate)<br>ii. Symptomatic pharyngitis (sore throat, GAS throat isolate)<br>iii. Acute rheumatic fever/Rheumatic heart disease	i. Skin<br>ii. Respiratory<br>iii. Cardiovascular	Mixed sources (self-reported and researcher collected)	Household crowding	5	Household crowding was extreme in communities 1 and 3 (10-21 people per household), where rates of infection were highest.	44
McDonald (2006)	Cohort	3 remote communities in the Top End<br>(NT)<br>MMM 7	Aboriginal or Torres Strait Islander	Mixed	i. Pyoderma (skin sores)<br>ii. Sore throat (GAS throat carriage)	i. Skin<br>ii. Respiratory	Researcher collected	Household crowding	5	There was no correlation between GAS throat carriage and household crowding, although there was a correlation between the prevalence of pyoderma and crowding in community 1	45

First author (publication date)	Type of study	Location / Setting (Jurisdiction) MMM	Population	Age category	Infectious disease (ID) outcomes			Exposures		General results	Ref
					ID as reported	Relevant ICPC2 body system	ID data source	As reported	HLP categories		
Melody (2016)	Cross-sectional	Remote communities<br>(WA)<br>MMM 7	Aboriginal or Torres Strait Islander	Not specified	i. Hearing issues<br>ii. Eyesight issues<br>iii. Asthma/respiratory issues<br>iv. Skin infection<br>v. Gastrointestinal infection/disease<br>vi. Mosquito borne health issues<br>vii. Kidney disease<br>viii. Flu/colds	i. Ear<br>ii. Eye<br>iii. Respiratory<br>iv. Skin<br>v. Digestive<br>vi. General<br>vii. Urological<br>viii. Respiratory	Linked data	Questions related to community infrastructure and physical environment; housing/overcrowding, dust, water quality/supply, rubbish removal	3, 5, 6, 7, 9, 10	Environmental factors/infrastructure issues were risk factors for certain health issues	46
Meloni (1993)	Cohort	Communities in the west Kimberley region<br>(WA)<br>MMM 7	Aboriginal or Torres Strait Islander	Mixed	i. Parasites- *Giardia duodenalis, Hymenolepsis nana, Entamoeba coli, Ancylostoma duodenale, Pentatrichomonas hominis, Chilomastix mesnili, Entamoeba hartmanni, Sarcocystis sp., Trichiuris trichiura, Enterobius vermicularis, Isospora belli, Strongyloides stercoralis*	i. Digestive	Researcher collected	Dogs and cats	6	Intestinal parasites common in children as well as dogs/cats	47
Memmott (2022)	Mixed method	Tennant Creek and Barkly Region<br>(NT)<br>MMM 7	Aboriginal or Torres Strait Islander	Mixed	i. Skin and skin structure infections (scabies, crusted scabies, skin sores, abscesses, carbuncles, furuncles [boils], wound infections, fungal infections, localised infections, impetigo)<br>ii. Respiratory infections (flu and flu-like illness, ear, nose and throat infections, otitis media [acute, suppurative, perforated, effusion], pharyngitis/sore throat including infection with GAS, ear canal infections [otitis externa], LRT and URT infections and exacerbations of chronic suppurative lung disease)<br>iii. Cardiac (including GAS manifestations- ARF/RHD)<br>iv. Optical (including trachoma and conjunctivitis)<br>v. Renal (including chronic kidney disease)<br>vi. Bone and joint infection<br>vii. Dental infection<br>viii. Hepatic infection<br>ix. Viral and bacterial infections<br>x. Gastrointestinal (including gastroenteritis and intestinal worms)	i. Skin<br>ii. Respiratory<br>iii. Cardiovascular<br>iv. Eye<br>v. Urological<br>vi. General<br>vii. General<br>viii. General<br>ix. General<br>x. Digestive	Mixed sources (self-reported and primary care)	Population of the household, housing use, functionality of health hardware, and services (including waste disposal); environmental health factors, including dust management and pet care, were also included.	1, 5, 6, 10		48

First author (publication date)	Type of study	Location / Setting (Jurisdiction) MMM	Population	Age category	Infectious disease (ID) outcomes			Exposures		General results	Ref
					ID as reported	Relevant ICPC2 body system	ID data source	As reported	HLP categories		
Merianos (1995)	Cohort	Alice Springs and Barkly Tablelands (NT), APY Lands (SA), Ngaanyatjarra Homelands (WA)<br>MMM 7	Aboriginal or Torres Strait Islander	Mixed	i. Gonococcal conjunctivitis	ii. Eye	Mixed sources (hospital admission data and notifiable disease registries)	Type of dwelling; household/family size; unwashed faces/hands; density of flies	1, 3, 5, 6	Association between washing hands/faces; explosive increase in fly density during epidemic	49
Milazzo (2017)	Cross-sectional	(SA)<br>MMM mixed	Not specified	Mixed	i. Campylobacter infection<br>ii. Salmonella infection	i. Digestive<br>ii. Digestive	Notifiable disease registries	Food safety knowledge and practices E.g. washing hands, chopping boards, food storage, defrosting, food transport etc.	4	Unsafe practices results in infection	50
Mishra (2023)	Model	National/focus on south-eastern Aust.<br>MMM mixed	Not specified	Not specified	i. Respiratory disease (lower respiratory tract infection, increasing severity of asthma/chronic obstructive pulmonary disease)	i. Respiratory	Modelled	Cold housing	8	Gains in health would be made by reducing cold housing	51
Moffatt (2020)	Cross-sectional	All jurisdictions except NSW<br>MMM mixed	Not specified	Mixed	i. *Campylobacter* sp. gastroenteritis	i. Digestive	Notifiable disease registries	Transmission routes related to food preparation, drinking water and animals	3, 4, 6	Outbreaks were foodborne (60%), waterborne (6%), animal-person (2.4%)	52
Murray-Smith (1996)	Case control	Charters Towers (Qld)<br>MMM 4	Not specified	Mixed	i. Dengue fever	i. General	Notifiable disease registries	Unscreened housing	6	Lack of screens facilitates spread of disease	53
Najnin (2014)	Cohort	(SA)<br>MMM mixed	Not specified	Mixed	i. Respiratory symptoms (sore throat, runny nose, cough)<br>ii. Dermal symptoms (rash, dermal infection, itching)<br>iii. Gastrointestinal symptom (loose stool or vomiting)	i. Respiratory<br>ii. Skin<br>iii. Digestive	Self-reported	Pet ownership; recreational swimming in any setting (related to faecal pathogens); number of people in the house	3, 5, 6		54
O'Toole (2012)	Cross-sectional	Melbourne (Vic)<br>MMM 1	Not specified	Mixed	i. Gastroenteritis/gastrointestinal illness (diarrhoea or vomiting)	i. Digestive	Self-reported	Greywater sampling	3	Overall strength of association was low	55
Oguoma (2023)	Cohort	National (11 sites in locations ranging from very remote communities to major capital cities)<br>MMM mixed	Aboriginal or Torres Strait Islander	Child/ adolescent (<12 y)	i. Otitis media (see list of symptoms)	i. Ear	Self-reported	Number of children and adults living in the same house; socio-economic advantage based on nine factors (2 related to housing) [not codified]	5	Large household sizes associated with OM	56
Peach (1997)	Cohort	Ballarat (Vic)<br>MMM 2	Not specified	Adult	i. *Helicobacter pylori* infection	i. Digestive	Researcher collected	Washing hands after toilet; household size; having ever lived on a farm	1, 5, 6	Related to household size and hand washing	57

First author (publication date)	Type of study	Location / Setting (Jurisdiction) MMM	Population	Age category	Infectious disease (ID) outcomes			Exposures		General results	Ref
					ID as reported	Relevant ICPC2 body system	ID data source	As reported	HLP categories		
Pearce (1995)	Cohort	Doomadgee (Qld) MMM 7	Aboriginal or Torres Strait Islander	Child (1-10 y)	i. Group C meningitis	i. General	Researcher collected	Poorly designed dwellings, small and in disrepair; large household sizes	5, 10		58
Potter (2016)	Qualitative	(WA) MMM mixed	Not specified	Adult	i. Mosquito-borne disease	i. General	Self-reported	Leaving water containers in the yard; maintaining pools, rainwater tanks and ponds; being bitten at home	3, 6		59
Ralph (2022)	Cohort	Three remote communities in northern Aust. (NT) MMM 7	Aboriginal or Torres Strait Islander	Mixed	i. Rheumatic heart disease<br>ii. Acute rheumatic fever (possible, probable, definite)<br>iii. Sore throat (Strep A pharyngitis/tonsillitis)<br>iv. Infected skin sore (impetigo) without abscesses<br>v. Scabies<br>vi. Skin boil<br>vii. Skin/soft tissue infection other than skin sores<br>viii. Ear infection<br>ix. Acute post-streptococcal glomerulonephritis<br>x. Fungal skin infection (tinea)<br>xi. Fever<br>xii. Lower respiratory tract infection<br>xiii. Upper respiratory tract infection	i. Cardiovascular<br>ii. Cardiovascular<br>iii. Respiratory<br>iv.Skin<br>v. Skin<br>vi. Skin<br>vii. Skin<br>viii. Ear<br>ix. Urological<br>x. Skin<br>xi. General<br>xii. Respiratory<br>xiii. Respiratory	Primary care	Household occupancy; bed sharing; presence and functionality of health hardware	1, 2, 3, 5	Associations with household size, sharing a mattress with others, unavailable soap, shower not working, not hot water in shower, toilet not working, no washing machine	60
Ratnaike (1989)	Cross-sectional	Yalata (SA) MMM 7	Aboriginal or Torres Strait Islander	Child (<5 y)	i. Diarrhoea	i. Digestive	Primary care	Preparing and consuming food by hand; lack of adequate storage and refrigeration facilities for food; comparisons of housing vs. camps	4, 10		61
Rodrigo (2011)	Random-ised controlled trial	Adelaide (SA) MMM 1	Not specified	Mixed	i. Highly credible gastroenteritis (characterized by a specified number of loose stools or vomiting alone or in combination with abdominal pain or nausea in a 24-hour period)	i. Digestive	Self-reported	Drinking water treatment	3	Consumption of untreated rainwater does not contribute to gastroenteritis	62
Schnagl (1978)	Cross-sectional	At least 7 towns across WA MMM mixed	Mixed	Mixed	i. Gastroenteritis/symptoms of diarrhoea	i. Digestive	Researcher collected	Specimens collected from dogs and other animals	6	Pleomorphic virus-like particles which resemble Coronaviruses found in both children and dogs, but equally present in those with or without symptoms.	63

First author (publication date)	Type of study	Location / Setting (Jurisdiction) MMM	Population	Age category	Infectious disease (ID) outcomes			Exposures		General results	Ref
					ID as reported	Relevant ICPC2 body system	ID data source	As reported	HLP categories		
Schrieber (2012)	Case study	Yarrabah (Qld) MMM 5	Aboriginal or Torres Strait Islander	Child	i. *Streptococcus dysgalactiae* subsp. *equisimilis*, also known as group G and C streptococci (throat infection)	i. Respiratory	Researcher collected	Swabs collected from dog	6	Transmission can occur between humans and dogs	64
Shattock (2014)	Model	Model reflecting a remote Aboriginal community (NA) MMM mixed	Aboriginal or Torres Strait Islander	Child/ adolescent (1-4, 5-14 y)	i. Trachoma	i. Eye	Modelled	Model simulated to reflect intervention efforts including housing development initiatives and improvements in prevalence of facial cleanliness	1, 10	Increased facial cleanliness and increased housing construction was found to increase the likelihood of controlling trachoma.	65
Sinclair (2010)	Case control	Suburbs served by the Rous water supply in Sydney (NSW) MMM 1	Not specified	Mixed	i. Gastroenteritis of presumed infectious origin<br>ii. Respiratory complaints of presumed infectious or allergic origin<br>iii. Dermal complaints of presumed infectious or allergic origin	i. Digestive<br>ii. Respiratory<br>iii. Skin	Primary care	Dual reticulation model; houses receive recycled water (from sewage) for toilet flushing and outdoor use and conventional water for other use (vs conventional model)	3	Little difference in number of medical consultations between residents of two water supply areas	66
Singleton (2014)	Cohort	(NT) MMM mixed	Aboriginal or Torres Strait Islander	Child/ adolescent (0.5-9 y)	i. Chronic suppurative lung disease/bronchiectasis (resulting from acute lower respiratory tract infections)<br>ii. Otitis media<br>iii. Gastroenteritis	i. Respiratory<br>ii. Ear<br>iii. Digestive	Researcher collected	Overcrowded housing; absence of a refrigerator; nutritional status; absence of running water; absence of flushing toilet	1, 3, 4, 5		67
Sordo (2022)	Cross-sectional	(NSW) MMM mixed	Not specified	Mixed	i. COVID-19	i. Respiratory	Notifiable disease registries	Household size/number of people in the house	5	No significant effect of household size on transmission	68
Speare (2003)	Cohort	Townsville (Qld) MMM 2	Not specified	Mixed	i. Head lice	i. Skin	Researcher collected	Pillowcase washing and drying	2	A reasonable strategy as part of treatment	69
Spurling (2013)	Cross-sectional	Brisbane (Qld) MMM 1	Aboriginal or Torres Strait Islander	Child/ adolescent (0-14 y)	i. Middle ear disease (bulging ear drums, wet perforation, dry perforation)	i. Ear	Researcher collected	Number of people living in the house at the time of the health check; presentation in winter months	5, 8	Middle ear disease in this study was associated with previous ear infection and increasing household number; presenting in a winter month	70
Tedesco (1980)	Cross-sectional	19 communities in 4 zones (NT) MMM 2	Aboriginal or Torres Strait Islander	Child/ adolescent (0-21 y)	i. Trachoma	i. Eye	Researcher collected	Diet/access to healthy food; water sources; toilet facilities; shower facilities; power; hot water; refrigerators; mangy dogs; sanitation; dusty and dehydrating environment	1, 2, 3, 4, 6, 7, 10	Incidence of trachoma related to the living conditions in each area.	71
Tellioglu (2022)	Model	Medium to large remote communities MMM 2	Aboriginal or Torres Strait Islander	Mixed	i. Scabies	i. Skin	Modelled	Household size and distribution	5	There is an effect of household size on scabies prevalence, especially age-specific	72

First author (publication date)	Type of study	Location / Setting (Jurisdiction) MMM	Population	Age category	Infectious disease (ID) outcomes			Exposures		General results	Ref
					ID as reported	Relevant ICPC2 body system	ID data source	As reported	HLP categories		
Tenkate (2001)	Case control	Brisbane Southside and South Coast areas (Qld) MMM 1	Not specified	Child (0-35 months)	i. Campylobacter infection	i. Digestive	Notifiable disease registries	Animal exposures, including types of household animals; food hygiene and food handling practices	4, 6	Ownership of dogs and chickens, and consumption of some foods independently associated with illness	73
Unicomb (2009)	Cohort	National MMM mixed	Not specified	Mixed	i. Campylobacter diseases	i. Digestive	Notifiable disease registries	Factors contributing to waterborne and foodborne disease	3, 4	Transmission mostly related to certain foods and practices.	74
Vino (2017)	Model	Royal Darwin Hospital servicing the Darwin region (NT) MMM mixed	Aboriginal or Torres Strait Islander	Adult (22-27 y)	i. Influenza-like illness	i. Respiratory	Modelled	Household crowding, household number, number of people who slept in each room	5	Household size and level of contact influences the peak outbreak time and overall affected population size	75
Williams (2015)	Case control	Darwin area (NT) MMM 2	Not specified	Child (0-4 y)	i. Salmonellosis	i. Digestive	Researcher collected	Samples collected from household environment to determine sources of infection; animal faeces; dogs, cats, rabbits, cockroach, rat, mouse etc; broom and vacuum cleaner dust samples; soil; kitchen samples	4, 6, 7, 9	Household environment is a source of salmonellosis among children in Darwin; vacuum cleaners and animal faeces important	76
Williams (2016)	Case study	Darwin and Palmerston (NT) MMM 2	Mixed	Child (0-4 y)	i. Salmonellosis	i. Digestive	Notifiable disease registries	Pet ownership and oral contact with animals; cleaning/sweeping; consuming certain foods; number of people in the household	4, 5, 6, 10	Sweeping in presence of children; contact with animals; consumption of powdered milk	77
Williams (2011)	Case study	Suburban Melbourne (Vic) MMM 2	Not specified	Mixed (>4 y)	i. Rickettsia felis	i. General	Hospital admission data	Kittens	6	Cases associated with family getting 2 kittens	78
Wong (2002)	Cohort	Wadeye (NT) MMM 7	Aboriginal or Torres Strait Islander	Child (<5 y)	i. Scabies<br>ii. Non-scabies pyoderma<br>iii. Infected scabies	i. Skin<br>ii. Skin<br>iii. Skin	Researcher collected	Community treatment/cleanup day. Residents cleaned their houses, washed their clothes/sheets and exposed to the sun their mattresses.	1, 2, 9	Program considered successful.	79
Wozniak (2022)	Cross-sectional	Remote communities in far north-WA, NT and far north Qld MMM 7	Not specified (although Aboriginal and Torres Strait	Not specified	i. Bloodstream infection<br>ii. Urinary tract infection<br>iii. Respiratory infection<br>iv. Skin and soft tissue infection (drug resistant bacteria: *Staphylococcus aureus*, *Escherichia coli*,	i. Blood<br>ii. Urological<br>iii. Respiratory<br>iv. Skin	Primary care	Average people per household; also used remoteness index	5	No association with household size. Remoteness is a risk factor for some AMR pathogens.	80

First author (publication date)	Type of study	Location / Setting (Jurisdiction) MMM	Population	Age category	Infectious disease (ID) outcomes			Exposures		General results	Ref
					**ID as reported**	**Relevant ICPC2 body system**	**ID data source**	**As reported**	**HLP categories**		
			Islander apparent focus)		*Pseudomonas aeruginosa*, *Klebsiella pneumoniae*)						
Wright (2022)	Cross-sectional	SE Qld MMM 1	Not specified	Mixed	i. Delta COVID-19 variant (SARS-CoV-2)	i. Respiratory	Notifiable disease registries	Number of bedrooms, bedroom sharing, bathrooms per household, additional household cleaning	1, 5	Less transmission in households with more bedrooms and bathrooms per person	81
Zajaczk-owski (2018)	Case control	SW Sydney (Bankstown, Camden, Campbelltown, Fairfield, Liverpool, Wingecarribee, Wollondilly) (NSW) MMM 1	Not specified	Mixed	i. Giardiasis	i. Digestive	Notifiable disease registries	Contact with farm animals, domestic animals or wildlife; household water storage and use	3, 6	Contact with animals was an identified risk factor	82

Table S7. Assessments of methodological quality of 73 articles included in the review that could be scored from a biomedical perspective using Joanna Briggs Institute (JBI) critical appraisal tools relevant to each study type [84]. Scores were marked as 1 for 'yes' or 'partial' and 0 for 'no', 'unclear' or 'not applicable'. The total number of questions in each tool varied between 8-13 depending on study type. The sum of 1's (yes scores) for each study was converted to a percentage of total possible yes scores for the relevant study design/tool; percentage scores are colour graded by quartiles and grouped within studies of the same design. Ten studies (models and mixed method designs) were not assessed as there were no relevant JBI tools. Articles with asterisks (*) were also scored against the Aboriginal and Torres Strait Islander Quality Appraisal Tool (Table S6).

First author surname	Year of publication	Type of study +JBI tool used	JBI Tool Question													Sum of 1's (yes)	% of total possible
			Q1	Q2	Q3	Q4	Q5	Q6	Q7	Q8	Q9	Q10	Q11	Q12	Q13		
Williams	2016	Case control	1	1	1	1	1	1	1	1	0	1				9	90
Tenkate	2001	Case control	1	1	0	1	1	1	0	1	1	1				8	80
Zajaczkowski	2018	Case control	1	1	0	1	1	1	1	1	0	1				8	80
Sinclair	2010	Case control	1	1	1	1	1	0	0	0	1	1				7	70
Murray-Smith	1996	Case control	1	1	1	0	1	0	0	0	0	1				5	50
Hempenstall*	2021	Case control	0	1	1	0	0	0	0	1	0	1				4	40
Williams	2015	Case control	0	0	0	1	1	0	0	1	0	1				4	40
Schrieber	2012	Case report	0	0	1	1	0	0	0	0						2	25
Williams	2011	Case report	0	0	1	1	0	0	0	0						2	25
McDonald*	2006	Cohort	1	1	1	1	1	0	1	1	1	1	1			10	91
Ewald*	2003	Cohort	1	1	1	0	0	1	1	1	1	1	1			9	82
Heyworth	2006	Cohort	1	1	1	1	1	0	0	1	1	1	1			9	82
La Vincente*	2009	Cohort	1	1	1	1	1	1	1	0	0	0	1			8	73
Leach*	2016	Cohort	1	1	1	0	0	0	1	1	1	1	1			8	73
Singleton	2014	Cohort	1	1	1	1	1	1	1	0	0	0	1			8	73
Andersen*	2018	Cohort	1	1	1	1	1	0	1	0	0	0	1			7	64
Bailie*	2005	Cohort	1	1	1	1	1	0	0	1	1	0	0			7	64
Hall	2017	Cohort	1	1	1	1	0	0	1	1	0	0	1			7	64
Harris*	1984	Cohort	1	1	1	1	1	1	1	0	0	0	0			7	64
Inglis*	1999	Cohort	1	1	1	1	0	0	0	0	1	1	1			7	64
McDonald*	2007	Cohort	1	1	1	0	0	0	1	1	1	0	1			7	64
Oguoma*	2023	Cohort	1	1	1	1	0	1	1	0	0	0	1			7	64
Peach	1997	Cohort	1	1	1	1	0	1	1	0	0	0	1			7	64
Unicomb	2009	Cohort	1	1	1	0	0	1	1	1	0	0	1			7	64
Harris*	1990	Cohort	1	1	1	1	0	0	0	1	1	0	0			6	55
Lansingh*	2010	Cohort	0	0	0	0	0	1	1	1	1	1	1			6	55
McDonald*	2008	Cohort	1	1	1	0	0	0	1	1	0	0	1			6	55
Merianos*	1995	Cohort	1	1	1	0	0	1	1	0	0	0	1			6	55
Najnin	2014	Cohort	1	1	1	0	0	1	1	0	0	0	1			6	55
Wong*	2002	Cohort	0	1	1	0	0	1	0	1	1	1	0			6	55
Jacoby*	2011	Cohort	1	1	1	1	0	0	0	0	1	0	0			5	45
Brown	2015	Cohort	1	1	0	0	0	0	1	0	0	0	1			4	36
Dossetor*	2017	Cohort	0	1	1	1	0	0	0	0	0	0	1			4	36
Meloni*	1993	Cohort	1	1	1	0	0	0	1	0	0	0	0			4	36
Ralph*	2022	Cohort	1	1	1	0	0	1	0	0	0	0	0			4	36

Boreham	1986	Cohort	1	1	1	0	0	0	0	0	0	0	0			3	**27**
May	2016	Cohort	0	0	1	0	0	0	1	0	0	0	1			3	**27**
Pearce	1995	Cohort	1	1	0	0	0	0	1	0	0	0	0			3	**27**
Speare	2003	Cohort	1	1	1	0	0	0	0	0	0	0	0			3	**27**
Cooper*	1986	Cohort	0	1	1	0	0	0	0	0	0	0	0			2	**18**
Edwards*	1970	Cohort	0	0	0	0	0	1	1	0	0	0	0			2	**18**
Bailie*	2012	Cross sectional	1	1	1	1	1	1	1	0						7	**88**
Bailie*	2010	Cross sectional	1	1	1	1	1	1	1	0						7	**88**
Milazzo	2017	Cross sectional	0	1	1	1	1	1	1	1						7	**88**
Carver	2008	Cross sectional	1	1	1	1	1	1	0	0						6	**75**
Heyworth	2003	Cross sectional	1	1	1	1	0	0	1	1						6	**75**
McBride	1998	Cross sectional	1	1	0	1	1	1	0	1						6	**75**
Spurling*	2013	Cross sectional	1	1	1	1	0	0	1	1						6	**75**
Bailie	2005	Cross sectional	1	1	1	1	0	0	1	0						5	**63**
Melody*	2016	Cross sectional	1	1	1	1	0	0	0	1						5	**63**
Sordo	2022	Cross sectional	1	1	1	0	0	0	1	1						5	**63**
Carcione	2011	Cross sectional	0	0	1	1	0	0	1	1						4	**50**
Hodgetts*	2022	Cross sectional	0	0	1	1	0	0	1	1						4	**50**
Looker	2010	Cross sectional	1	1	1	1	0	0	0	0						4	**50**
Marshall	2011	Cross sectional	0	1	1	1	0	0	1	0						4	**50**
Tedesco*	1980	Cross sectional	0	1	0	1	0	0	1	1						4	**50**
Wright	2022	Cross sectional	1	1	1	1	0	0	0	0						4	**50**
Akter	2017	Cross sectional	0	1	0	1	0	0	0	1						3	**38**
Hanna*	1996	Cross sectional	0	1	0	1	0	0	1	0						3	**38**
Chen	2014	Cross sectional	0	0	0	0	1	1	0	0						2	**25**
Edwards*	1970	Cross sectional	0	0	0	1	0	0	1	0						2	**25**
Kaminski*	1977	Cross sectional	0	1	0	1	0	0	0	0						2	**25**
Moffatt	2020	Cross sectional	0	0	1	1	0	0	0	0						2	**25**
O'Toole	2012	Cross sectional	1	0	1	0	0	0	0	0						2	**25**
Ratnaike*	1989	Cross sectional	0	1	0	0	0	1	0	0						2	**25**
Schnagl	1978	Cross sectional	0	1	0	1	0	0	0	0						2	**25**
Wozniak	2022	Cross sectional	0	0	0	1	0	0	1	0						2	**25**
Andersen*	2016	Qualitative	1	1	1	1	1	1	1	1	1	1				10	**100**
Chakraborty	2021	Qualitative	1	1	1	1	1	1	1	1	1	1				10	**100**
Kerrigan*	2021	Qualitative	1	1	1	1	1	1	1	1	1	1				10	**100**
Potter	2016	Qualitative	1	1	1	1	1	0	1	0	0	1				7	**70**
Massey*	2009	Qualitative	1	1	1	0	1	0	0	1	0	1				6	**60**
Rodrigo	2011	RCT	0	1	1	1	1	1	1	0	1	1	1	1	0	10	**77**

Notes: For case-control studies, JBI scores ranged from 40-90%; scores were commonly reduced due to lack of clarity as to whether exposures were long enough to be meaningful, and failure to identify confounding factors. Two case reports described clinical conditions of interest but failed to clearly report on the patient's history, the intervention and the takeaway lessons, so both scored 25%. Scores for cross-sectional studies ranged from 25-88%; scores were mostly reduced because criteria for inclusion in the sample were not clearly defined, and confounding factors were not discussed. The single RCT scored 77% against the relevant JBI tool [62]; scores were reduced as true randomisation was not used, and groups lost to follow up were not adequately described. Three qualitative studies scored

100% whilst scores for other qualitative studies were reduced as there was uncertainty surrounding the positionality of the authors and the adequate representation of participant voices.

Table S8. Assessments of methodological quality of 40 studies involving Indigenous people and communities that could be scored from an Indigenous cultural perspective. Scores were marked as 1 for 'yes' or 'partial' and 0 for 'no' or 'unclear' against 14 questions set out in the Aboriginal and Torres Strait Islander Quality Appraisal Tool (QAT) [83]. The sum of 1's (yes scores) for each study was converted to a percentage of 14 total possible yes scores; percentage scores are colour graded by quartiles. Articles with asterisks (*) were also scored against relevant JBI tools (Table S5).

First author surname	Year of publication	Aboriginal and Torres Strait Islander QAT Question														Sum of 1s (yes or partial)	% of total possible (14)
		Q1	Q2	Q3	Q4	Q5	Q6	Q7	Q8	Q9	Q10	Q11	Q12	Q13	Q14		
Kerrigan*	2021	1	1	1	1	1	0	0	1	1	1	1	1	1	1	12	86
La Vincente*	2009	1	1	1	1	1	0	0	1	1	1	1	1	1	1	12	86
McDonald	2010	1	1	1	1	1	0	0	1	1	1	1	1	1	1	12	86
McDonald *	2007	1	1	1	1	1	0	0	1	1	1	1	1	1	1	12	86
Ralph*	2022	1	1	1	1	1	0	0	1	1	1	1	1	1	1	12	86
Dossetor*	2017	1	1	1	1	1	0	0	1	1	1	0	1	1	1	11	79
Memmott	2022	1	1	1	1	1	0	0	1	1	1	0	1	1	1	11	79
Ratnaike*	1989	1	1	1	1	0	1	1	1	1	1	1	0	1	0	11	79
Andersen*	2016	1	1	1	1	1	0	0	1	1	1	1	0	0	1	10	71
Ewald*	2003	1	1	1	0	1	0	0	1	1	1	1	1	1	0	10	71
Hui*	2021	1	1	1	1	0	0	0	1	1	1	1	1	0	1	10	71
Bailie*	2012	1	1	1	0	1	0	0	1	0	0	1	1	1	1	9	64
Lansingh*	2010	0	1	1	1	1	0	0	1	0	1	0	1	1	1	9	64
Wong*	2002	0	1	1	0	1	0	0	0	1	1	1	1	1	1	9	64
Bailie*	2010	1	1	1	0	1	0	0	1	0	0	1	0	1	1	8	57
Andersen*	2018	1	1	1	1	0	0	0	1	0	0	1	0	1	1	8	57
Massey*	2009	1	1	1	1	1	0	0	1	0	1	1	0	0	0	8	57
McDonald*	2008	0	1	1	1	1	0	0	1	0	0	0	1	1	1	8	57
Bailie*	2005	1	1	1	1	0	0	0	1	0	0	1	0	1	0	7	50
Shattock	2014	0	0	1	1	0	0	0	1	1	1	1	0	1	0	7	50
McDonald*	2009	1	1	0	1	1	0	0	1	0	0	0	0	0	0	5	36
McDonald*	2006	0	1	1	1	1	0	0	1	0	0	0	0	0	0	5	36
Foster	2021	1	0	0	0	0	0	0	0	0	0	1	1	0	0	3	21
Jacoby*	2011	**0**	**1**	**0**	**1**	**0**	**0**	**0**	**0**	**0**	**0**	**0**	**0**	**0**	**1**	3	21
Leach*	2016	0	1	0	1	0	0	0	1	0	0	0	0	0	0	3	21
Hanna*	1996	1	0	0	0	0	0	0	0	0	0	1	0	0	1	3	21
Cooper*	1986	1	0	0	0	0	0	0	0	0	0	0	1	0	0	2	14
Edwards*	1970	0	0	0	0	0	0	0	0	0	0	0	1	1	0	2	14
Harris*	1990	1	0	1	0	0	0	0	0	0	0	0	0	0	0	2	14
Oguoma*	2023	0	1	0	0	0	0	0	0	0	0	0	1	0	0	2	14
Hempenstall*	2021	0	0	0	0	0	0	0	0	0	0	0	1	0	0	1	7
Spurling*	2013	0	0	0	0	0	0	0	1	0	0	0	0	0	0	1	7
Inglis*	1999	0	0	0	0	0	0	0	1	0	0	0	0	0	0	1	7
Tedesco*	1980	1	0	0	0	0	0	0	0	0	0	0	0	0	0	1	7

Harris*	1984	0	0	0	0	0	0	0	0	0	0	0	0	0	0	0	0
Hodgetts*	2022	0	0	0	0	0	0	0	0	0	0	0	0	0	0	0	0
Kaminski*	1977	0	0	0	0	0	0	0	0	0	0	0	0	0	0	0	0
Melody*	2016	0	0	0	0	0	0	0	0	0	0	0	0	0	0	0	0
Meloni*	1993	0	0	0	0	0	0	0	0	0	0	0	0	0	0	0	0
Merianos*	1995	0	0	0	0	0	0	0	0	0	0	0	0	0	0	0	0
**Total**		**22**	**24**	**22**	**20**	**17**	**1**	**1**	**24**	**13**	**15**	**18**	**18**	**18**	**17**		
**% (out of 40 studies)**		**55**	**60**	**55**	**50**	**43**	**3**	**3**	**60**	**33**	**38**	**45**	**45**	**45**	**43**		

Notes: Of the 40 studies eligible for appraisal against the Aboriginal and Torres Strait Islander QAT, almost half (*n*=18) scored less than 25% including six studies that scored 0%; poorly scoring studies were mostly published prior to the year 2000. The most common reasons for reduced scores surrounded data sovereignty; clear agreements regarding access and control over intellectual and cultural property were rarely negotiated (Q6 and Q7). In most cases (more than half of studies), the research did respond to a need or priority determined by the community (Q1), community consultation was appropriately inclusive (Q2), and there was demonstrated community control over data collection (Q8). However, in most cases (more than half of studies) there was no clear benefit to the participants or community, and no evidence for capacity building embedded in the research (Q12 and Q13). It was generally difficult to determine whether Indigenous leadership and governance were genuine and meaningful (Q3, Q4), and whether the research was guided by an Indigenous research paradigm (Q9). The Aboriginal and Torres Strait Islander QAT could not be applied to models, except for Hui et al.[28], which scored 71%; ongoing partnership with an Indigenous Advisory Group was a strength of this study compared to other model-based studies, but data sovereignty regarding model inputs remained difficult to define and resulted in reduced scores (Q6 and Q7).

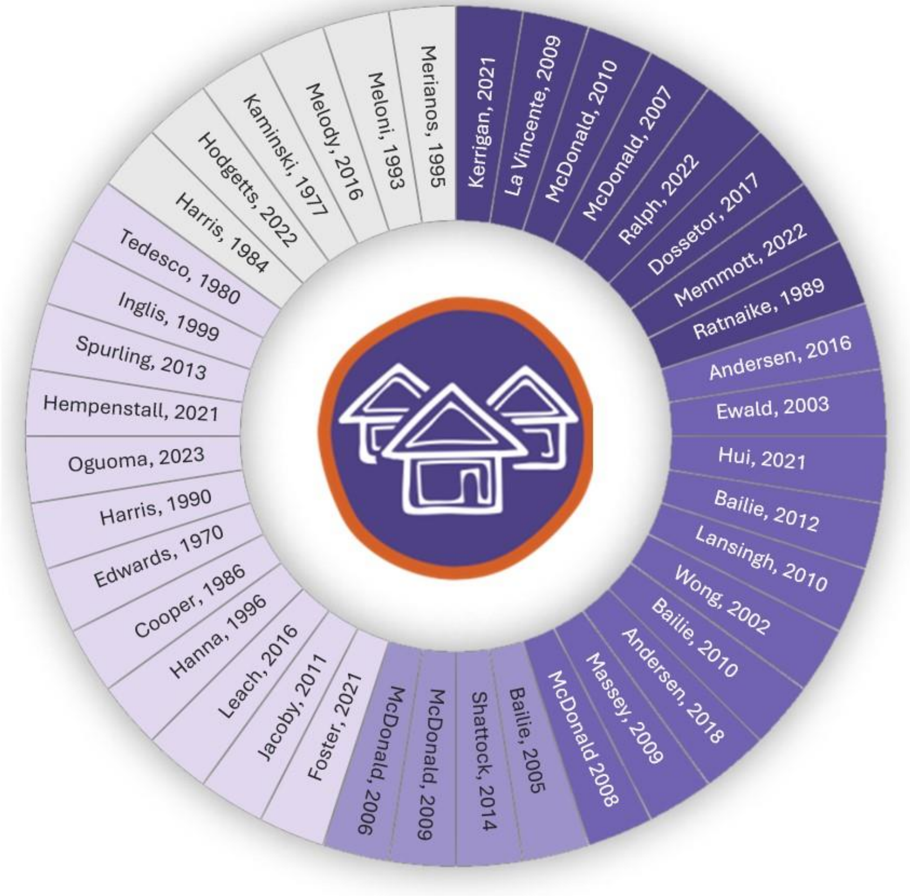

Figure S2. Assessments of methodological quality from an Indigenous cultural perspective, scored using the Aboriginal and Torres Strait Islander QAT. Increased intensity of shading indicates higher quality.

The application of the Aboriginal and Torres Strait Islander QAT was challenging for our team. We wanted to ensure that quantitative biomedical approaches to methodological quality appraisal were companioned by Indigenous approaches. However, the reality may be that applying numerical scores to discrete questions does not necessarily reflect Aboriginal and Torres Strait Islander ways of knowing, being and doing, or of culturally sound research. Applying the scoring guide felt reductive and did not always produce scores congruent with the feeling of Aboriginal co-authors. There was little opportunity to reflect the nuance and complexity of working well in this space. Aboriginal co-authors preferred to visualise scores in a way that reflected the sense of the article (for example, in Cai and colleagues[85]). Cultural safety and quality standards in research have developed rapidly in the years since the introduction of the Aboriginal and Torres Strait Islander QAT (2020) and particularly in the increasing use of the Consolidated Criteria for Strengthening Reporting of Health Research Involving Indigenous Peoples (the CONSIDER statement, 2019). This meant that more recent studies reported on cultural considerations in greater detail; it is unclear whether this reflects improvement in the way research is conducted or merely in the way it is reported. Overall, the Aboriginal and Torres Strait Islander QAT was a

useful prompt for considering cultural aspects but an imperfect vehicle for representing a holistic sense of individual publications and their relative values.

References

1. Page MJ, McKenzie JE, Bossuyt PM, Boutron I, Hoffmann TC, Mulrow CD, Shamseer L, Tetzlaff JM, Akl EA, Brennan SE. The PRISMA 2020 statement: an updated guideline for reporting systematic reviews. *BMJ* 2021; 372.
2. Akter R, Naish S, Hu W, Tong S. Socio-demographic, ecological factors and dengue infection trends in Australia. *PLoS One*. 2017; 12(10):e0185551.
3. Andersen MJ, Williamson AB, Fernando P, Redman S, Vincent F. "There's a housing crisis going on in Sydney for Aboriginal people": focus group accounts of housing and perceived associations with health. *BMC Public Health*. 2016; 16(1):429.
4. Andersen MJ, Skinner A, Williamson AB, Fernando P, Wright D. Housing conditions associated with recurrent gastrointestinal infection in urban Aboriginal children in NSW, Australia: findings from SEARCH. *Aust N Z J Public Health*. 2018; 42(3):247-253.
5. Bailie RS, Stevens M, McDonald EL. The impact of housing improvement and socio-environmental factors on common childhood illnesses: a cohort study in Indigenous Australian communities. *J Epidemiol Public Health*. 2012; 66(9):821-831.
6. Bailie RS, Stevens MR, McDonald E, Halpin S, Brewster D, Robinson G, Guthridge S. Skin infection, housing and social circumstances in children living in remote Indigenous communities: testing conceptual and methodological approaches. *BMC Public Health*. 2005; 5(1):128.
7. Bailie R, Stevens M, McDonald E, Brewster D, Guthridge S. Exploring cross-sectional associations between common childhood illness, housing and social conditions in remote Australian Aboriginal communities. *BMC Public Health*. 2010; 10:1-10.
8. Boreham PFL, Phillips RE. Giardiasis in Mount Isa, north-west Queensland. *Med J Aust*. 1986; 144(10):524-528.
9. Brown CR, McCaw JM, Fairmaid EJ, Brown LE, Leder K, Sinclair M, McVernon J. Factors associated with transmission of influenza-like illness in a cohort of households containing multiple children. *Influenza Other Respir Viruses*. 2015; 9(5):247-254.
10. Carcione D, Giele CM, Goggin LS, Kwan KS, Smith DW, Dowse GK, Mak DB, Effler P. Secondary attack rate of pandemic influenza A(H1N1) 2009 in Western Australian households, 29 May-7 August 2009. *Euro Surveill*. 2011; 16(3).
11. Carver S, Sakalidis V, Weinstein P. House mouse abundance and Ross River virus notifications in Victoria, Australia. *Int J Infect Dis*. 2008; 12(5):528-533.
12. Chakraborty A, Howard NJ, Daniel M, Chong A, Slavin N, Brown A, Cargo M. Prioritizing built environmental factors to tackle chronic and infectious diseases in remote Northern Territory (NT) communities of Australia: a concept mapping study. *Int J Env Res Public Health*. 2021; 18(10).
13. Chen Y, Williams E, Kirk M. Risk factors for acute respiratory infection in the Australian community. *PLoS One*. 2014; 9(7):e101440.
14. Chisholm RH, Crammond B, Wu Y, Bowen AC, Campbell PT, Tong SYC, McVernon J, Geard N. A model of population dynamics with complex household structure and mobility: implications for transmission and control of communicable diseases. *PeerJ*. 2020; 8:e10203.
15. Cooper RL, Coid D, Constable IJ. Trachoma: 1985 update in Western Australia. *Aust N Z J Ophthalmol*. 1986; 14(4):319-323.
16. Dossetor PJ, Martiniuk ALC, Fitzpatrick JP, Oscar J, Carter M, Watkins R, Elliott EJ, Jeffery HE, Harley D. Pediatric hospital admissions in Indigenous children: a population-based study in remote Australia. *BMC Pediatr*. 2017; 17(1):195.
17. Edwards LD. Malnutrition and disease in pre-school Aboriginal children in the Walgett area of N.S.W. *Med J Aust*. 1970; 2(22):1007-1012.
18. Ewald DP, Hall GV, Franks CC. An evaluation of a SAFE-style trachoma control program in central Australia. *Med J Aust*. 2003; 178(2):65-68.
19. Foster T, Hall NL. Housing conditions and health in Indigenous Australian communities: current status and recent trends. *Int J Environ Health Res*. 2021; 31(3):325-343.
20. Hall KK, Chang AB, Anderson J, Arnold D, Goyal V, Dunbar M, Otim M, O'Grady KF. The Incidence and short-term outcomes of acute respiratory illness with cough in children from a socioeconomically disadvantaged urban community in Australia: a community-based prospective cohort study. *Front Pediatr*. 2017; 5:228.

21. Hanna JN, Ritchie SA, Phillips DA, Shield J, Bailey MC, Mackenzie JS, Poidinger M, McCall BJ, Mills PJ. An outbreak of Japanese encephalitis in the Torres Strait, Australia, 1995. *Med J Aust*. 1996; 165(5):256-260.
22. Harris MF, Kamien M. Change in Aboriginal childhood morbidity and mortality in Bourke 1971-84. *J Paediatr Child Health*. 1990; 26(2):80-84.
23. Harris MF, Nolan B, Davidson A. Early childhood pneumonia in Aborigines of Bourke, New South Wales. *Med J Aust*. 1984; 140(12):705-707.
24. Hempenstall A, Howell E, Kang K, Chau KWT, Browne A, Kris E, Wapau H, Pilot P, Smith S, Reeves B *et al*. Echocardiographic screening detects a significant burden of rheumatic heart disease in Australian Torres Strait Islander children and missed opportunities for its prevention. *Am J Trop Med Hyg*. 2021; 104(4):1211-1214.
25. Heyworth JS, Baghurst P, McCaul KA. Prevalence of gastroenteritis among 4-year-old children in South Australia. *Epidemiol Infect*. 2003; 130(3):443-451.
26. Heyworth JS, Glonek G, Maynard EJ, Baghurst PA, Finlay-Jones J. Consumption of untreated tank rainwater and gastroenteritis among young children in South Australia. *Int J Epidemiol*. 2006; 35(4):1051-1058.
27. Hodgetts K, Kleinecke M, Woerle C, Kaestli M, Budd R, Webb JR, Ward L, Mayo M, Currie BJ, Meumann EM. Melioidosis in the remote Katherine region of northern Australia. *PLoS Negl Trop Dis*. 2022; 16(6):e0010486.
28. Hui BB, Brown D, Chisholm RH, Geard N, McVernon J, Regan DG. Modelling testing and response strategies for COVID-19 outbreaks in remote Australian Aboriginal communities. *BMC Infect Dis*. 2021; 21(1):929.
29. Inglis TJ, Garrow SC, Adams C, Henderson M, Mayo M, Currie BJ. Acute melioidosis outbreak in Western Australia. *Epidemiol Infect*. 1999; 123(3):437-443.
30. Jacoby P, Carville KS, Hall G, Riley TV, Bowman J, Leach AJ, Lehmann D. Crowding and other strong predictors of upper respiratory tract carriage of otitis media-related bacteria in Australian Aboriginal and non-Aboriginal children. *Pediatr Infect Dis J*. 2011; 30(6):480-485.
31. Kaminski GW, Green AC. Tinea capitis in Aboriginal children at Maningrida, Northern Territory, Australia. A variant of *Microsporum canis*. *Australas J Dermatol*. 1977; 18(2):88-97.
32. Kerrigan V, Kelly A, Lee AM, Mungatopi V, Mitchell AG, Wyber R, Ralph AP. A community-based program to reduce acute rheumatic fever and rheumatic heart disease in northern Australia. *BMC Health Services Res*. 2021; 21(1):1127.
33. La Vincente S, Kearns T, Connors C, Cameron S, Carapetis J, Andrews R. Community management of endemic scabies in remote aboriginal communities of northern Australia: low treatment uptake and high ongoing acquisition. *PLoS Negl Trop Dis*. 2009; 3(5):e444.
34. Lansingh VC, Mukesh BN, Keeffe JE, Taylor HR. Trachoma control in two central Australian Aboriginal communities: a case study. *Int Ophthalmol*. 2010; 30(4):367-375.
35. Leach AJ, Wigger C, Beissbarth J, Woltring D, Andrews R, Chatfield MD, Smith-Vaughan H, Morris PS. General health, otitis media, nasopharyngeal carriage and middle ear microbiology in Northern Territory Aboriginal children vaccinated during consecutive periods of 10-valent or 13-valent pneumococcal conjugate vaccines. *Int J Pediatr Otorhinolaryngol*. 2016; 86:224-232.
36. Looker C, Carville K, Grant K, Kelly H. Influenza A (H1N1) in Victoria, Australia: a community case series and analysis of household transmission. *PLoS One*. 2010; 5(10):e13702.
37. Marshall HM, Carter R, Torbey MJ, Minion S, Tolson C, Sidjabat HE, Huygens F, Hargreaves M, Thomson RM. *Mycobacterium lentiflavum* in drinking water supplies, Australia. *Emerg Infect Dis*. 2011; 17(3):395-402.
38. Massey PD, Pearce G, Taylor KA, Orcher L, Saggers S, Durrheim DN. Reducing the risk of pandemic influenza in Aboriginal communities. *Rural Remote Health*. 2009; 9(3):1290.
39. May FJ, Polkinghorne BG, Fearnley EJ. Epidemiology of bacterial toxin-mediated foodborne gastroenteritis outbreaks in Australia, 2001 to 2013. *Commun Dis Intell Q Rep*. 2016; 40(4):E460-E469.
40. McBride WJ, Mullner H, Muller R, Labrooy J, Wronski I. Determinants of dengue 2 infection among residents of Charters Towers, Queensland, Australia. *Am J Epidemiol*. 1998; 148(11):1111-1116.
41. McDonald E, Bailie R, Grace J, Brewster D. A case study of physical and social barriers to hygiene and child growth in remote Australian Aboriginal communities. *BMC Public Health*. 2009; 9(1):346.
42. McDonald E, Bailie R, Grace J, Brewster D. An ecological approach to health promotion in remote Australian Aboriginal communities. *Health Promotion Int*. 2010; 25(1):42-53.

43. McDonald M, Brown A, Edwards T, Hope A, Amu M, Morey F, Currie BJ, Carapetis JR. Apparent contrasting rates of pharyngitis and pyoderma in regions where rheumatic heart disease is highly prevalent. *Heart Lung Circ*. 2007; 16(4):254-259.
44. McDonald MI, Towers RJ, Andrews R, Benger N, Fagan P, Currie BJ, Carapetis JR. The dynamic nature of group A streptococcal epidemiology in tropical communities with high rates of rheumatic heart disease. *Epidemiol Infect*. 2008; 136(4):529-539.
45. McDonald MI, Towers RJ, Andrews RM, Benger N, Currie BJ, Carapetis JR. Low rates of streptococcal pharyngitis and high rates of pyoderma in Australian Aboriginal communities where acute rheumatic fever is hyperendemic. *Clin Infect Dis*. 2006; 43(6):683-689.
46. Melody SM, Bennett E, Clifford HD, Johnston FH, Shepherd CCJ, Alach Z, Lester M, Wood LJ, Franklin P, Zosky GR. A cross-sectional survey of environmental health in remote Aboriginal communities in Western Australia. *Int J Env Health Res*. 2016; 26(5-6):525-535.
47. Meloni BP, Thompson RC, Hopkins RM, Reynoldson JA, Gracey M. The prevalence of giardia and other intestinal parasites in children, dogs and cats from aboriginal communities in the Kimberley. *Med J Aust*. 1993; 158(3):157-159.
48. Memmott P, Lansbury N, Go-Sam C, Nash D, Redmond AM, Barnes S, Simpson P, Frank PN. Aboriginal social housing in remote Australia: crowded, unrepaired and raising the risk of infectious diseases. *Global Discourse*. 2022; 12(2):255-284.
49. Merianos A, Condon RJ, Tapsall JW, Jayathissa S, Mulvey G, Lane JM, Patel MS, Rouse I. Epidemic gonococcal conjunctivitis in central Australia. *Med J Aust*. 1995; 162(4):178-181.
50. Milazzo A, Giles LC, Zhang Y, Koehler AP, Hiller JE, Bi P. Factors influencing knowledge, food safety practices and food preferences during warm weather of salmonella and campylobacter cases in South Australia. *Foodborne Pathog Dis*. 2017; 14(3):125-131.
51. Mishra SR, Wilson T, Andrabi H, Ouakrim DA, Li A, Akpan E, Bentley R, Blakely T. The total health gains and cost savings of eradicating cold housing in Australia. *Social Sci Med*. 2023; 334(ut9, 8303205):115954.
52. Moffatt CRM, Fearnley E, Bell R, Wright R, Gregory J, Sloan-Gardner T, Kirk M, Stafford R. Characteristics of Campylobacter gastroenteritis outbreaks in Australia, 2001 to 2016. *Foodborne Pathog Dis*. 2020; 17(5):308-315.
53. Murray-Smith S, Weinstein P, Skelly C. Field epidemiology of an outbreak of dengue fever in Charters Towers, Queensland: Are insect screens protective? *Aust N Z J Public Health*. 1996; 20(5):545-547.
54. Najnin N, Forbes A, Sinclair M, Leder K. Risk factors for community-based reports of gastrointestinal, respiratory, and dermal symptoms: findings from a cohort study in Australia. *J Epidemiol*. 2014; 24(1):39-46.
55. O'Toole J, Sinclair M, Malawaraarachchi M, Hamilton A, Barker SF, Leder K. Microbial quality assessment of household greywater. *Water Res*. 2012; 46(13):4301-4313.
56. Oguoma VM, Mathew S, Begum T, Dyson E, Ward J, Leach AJ, Barzi F. Trajectories of otitis media and association with health determinants among Indigenous children in Australia: the longitudinal study of Indigenous children. *Public Health*. 2023; 225(qi7, 0376507):53-62.
57. Peach HG. *Helicobacter pylori* infection in an Australian regional city: prevalence and risk factors. *Med J Aust*. 1997; 167(6):310-313.
58. Pearce MC, Sheridan JW, Jones DM, Lawrence GW, Murphy DM, Masutti B, McCosker C, Douglas V, George D, O'Keefe A. Control of group C meningococcal disease in Australian Aboriginal children by mass rifampicin chemoprophylaxis and vaccination. *Lancet (London, England)*. 1995; 346(8966):20-23.
59. Potter A, Jardine A, Neville PJ. A survey of knowledge, attitudes, and practices in relation to mosquitoes and mosquito-borne disease in Western Australia. *Front Public Health*. 2016; 4:32.
60. Ralph AP, Kelly A, Lee AM, Mungatopi VL, Babui SR, Budhathoki NK, Wade V, Dassel JL, Wyber R. Evaluation of a community-led program for primordial and primary prevention of rheumatic fever in remote northern Australia. *Int J Environ Res Public Health*. 2022; 19(16).
61. Ratnaike RN, Ratnaike SK. Diarrhoeal disease in under five year olds: an epidemiological study in an Australian Aboriginal community. *Eur J Epidemiol*. 1989; 5(1):82-86.
62. Rodrigo S, Sinclair M, Forbes A, Cunliffe D, Leder K. Drinking rainwater: a double-blinded, randomized controlled study of water treatment filters and gastroenteritis incidence. *Am J Public Health*. 2011; 101(5):842-847.
63. Schnagl RD, Holmes IH, Mackay-Scollay EM. Coronavirus-like particles in Aboriginals and non-Aboriginals in Western Australia. *Med J Aust*. 1978; 1(6):307-309.

64. Schrieber L, Towers R, Muscatello G, Speare R. Transmission of *Streptococcus dysgalactiae* subsp. *equisimilis* between child and dog in an Aboriginal Australian community. *Zoonoses Public Health*. 2014; 61(2):145-148.
65. Shattock AJ, Gambhir M, Taylor HR, Cowling CS, Kaldor JM, Wilson DP. Control of trachoma in Australia: a model based evaluation of current interventions. *PLoS Neg Trop Dis*. 2015; 9(4).
66. Sinclair M, O'Toole J, Forbes A, Carr D, Leder K. Health status of residents of an urban dual reticulation system. *Int J Epidemiol*. 2010; 39(6):1667-1675.
67. Singleton RJ, Valery PC, Morris P, Byrnes CA, Grimwood K, Redding G, Torzillo PJ, McCallum G, Chikoyak L, Mobberly C *et al*. Indigenous children from three countries with non-cystic fibrosis chronic suppurative lung disease/bronchiectasis. *Pediatr Pulmonol*. 2014; 49(2):189-200.
68. Sordo AA, Dunn A, Gardiner ER, Reinten TA, Tsang TS, Deng L, Liu BC. Household transmission of COVID-19 in 2020 in New South Wales, Australia. *Comm Dis Intell*. 2022; 46.
69. Speare R, Cahill C, Thomas G. Head lice on pillows, and strategies to make a small risk even less. *Int J Dermatol*. 2003; 42(8):626-629.
70. Spurling GK, Askew DA, Schluter PJ, Simpson F, Hayman NE. Household number associated with middle ear disease at an urban Indigenous health service: a cross-sectional study. *Aust J Prim Health*. 2014; 20(3):285-290.
71. Tedesco LR. Trachoma and environment in the Northern Territory of Australia. *Soc Sci Med*. 1980; 14(2):111-117.
72. Tellioglu N, Chisholm RH, McVernon J, Geard N, Campbell PT. The efficacy of sampling strategies for estimating scabies prevalence. *PLoS Neg Trop Dis*. 2022; 16(6).
73. Tenkate TD, Stafford RJ. Risk factors for campylobacter infection in infants and young children: a matched case-control study. *Epidemiol Infect*. 2001; 127(3):399-404.
74. Unicomb LE, Fullerton KE, Kirk MD, Stafford RJ. Outbreaks of campylobacteriosis in Australia, 2001 to 2006. *Foodborne Pathog Dis*. 2009; 6(10):1241-1250.
75. Vino T, Singh GR, Davison B, Campbell PT, Lydeamore MJ, Robinson A, McVernon J, Tong SYC, Geard N. Indigenous Australian household structure: A simple data collection tool and implications for close contact transmission of communicable diseases. *PeerJ*. 2017; 5:e3958.
76. Williams S, Patel M, Markey P, Muller R, Benedict S, Ross I, Heuzenroeder M, Davos D, Cameron S, Krause V. Salmonella in the tropical household environment- Everyday, everywhere. *J Infect*. 2015; 71(6):642-648.
77. Williams S, Markey P, Harlock M, Binns P, Gaggin J, Patel M. Individual and household-level risk factors for sporadic salmonellosis in children. *J Infect*. 2016; 72(1):36-44.
78. Williams M, Izzard L, Graves SR, Stenos J, Kelly JJ. First probable Australian cases of human infection with Rickettsia felis (cat-flea typhus). *Med J Aust*. 2011; 194(1):41-43.
79. Wong LC, Amega B, Barker R, Connors C, Dulla ME, Ninnal A, Cumaiyi MM, Kolumboort L, Currie BJ. Factors supporting sustainability of a community-based scabies control program. *Australas J Dermatol*. 2002; 43(4):274-277.
80. Wozniak TM, Cuningham W, Ledingham K, McCulloch K. Contribution of socio-economic factors in the spread of antimicrobial resistant infections in Australian primary healthcare clinics. *J Glob Antimicrob Resist*. 2022; 30:294-301.
81. Wright E, Pollard G, Robertson H, Anuradha S. Household transmission of the Delta COVID-19 variant in Queensland, Australia: a case series. *Epidemiol Infect*. 2022; 150:e173.
82. Zajaczkowski P, Mazumdar S, Conaty S, Ellis JT, Fletcher-Lartey SM. Epidemiology and associated risk factors of giardiasis in a peri-urban setting in New South Wales Australia. *Epidemiol Infect*. 2019; 147.
83. Harfield S, Pearson O, Morey K, Kite E, Canuto K, Glover K, Gomersall JS, Carter D, Davy C, Aromataris E *et al*. Assessing the quality of health research from an Indigenous perspective: the Aboriginal and Torres Strait Islander quality appraisal tool. *BMC Med Res Methodol*. 2020; 20(1):79.
84. JBI. Critical appraisal tools. [https://jbi.global/critical-appraisal-tools]. Accessed March, 2025.
85. Cai Y, Delaney LJ, Lynch D, Cunninghame J, Takashima M, Ullman A, Toombs M, Martin A, Thompson K, Maxwell K *et al*. Ending rheumatic heart disease in Aboriginal and Torres Strait Islander communities: a systematic review of prevention programs in Australia. *Lowitja J*. 2025; 3:100040.
86. Huria T, Palmer SC, Pitama S, Beckert L, Lacey C, Ewen S, Smith LT. Consolidated criteria for strengthening reporting of health research involving Indigenous peoples: the CONSIDER statement. *BMC Med Res Methodol*. 2019; 19(1):173.
